# Generation of mitochondria-rich kidney organoids from expandable intermediate mesoderm progenitors reprogrammed from human urine cells under defined medium

**DOI:** 10.1186/s13578-022-00909-0

**Published:** 2022-10-15

**Authors:** Yapei Yuan, Huan Chen, Sihua Ou, Baomei Cai, Ruifang Zhang, Yue Qin, Mengjie Pan, Shangtao Cao, Duanqing Pei, Fan Fan Hou

**Affiliations:** 1 Division of Nephrology, National Clinical Research Center for Kidney Disease, State Key Laboratory of Organ Failure Research, Guangdong Provincial Key Laboratory of Renal Failure Research, Nanfang Hospital, Southern Medical University, Guangdong Provincial Institute of Nephrology, Guangzhou, 510515 China; 2 Bioland Laboratory (Guangzhou Regenerative Medicine and Health Guangdong Laboratory), Guangzhou, 510005 China; 3 Laboratory of Cell Fate Control, School of Life Sciences, Westlake University, Hangzhou, 310024 China; 4 CAS Key Laboratory of Regenerative Biology, Guangdong Provincial Key Laboratory of Stem Cell and Regenerative Medicine, Guangzhou Institutes of Biomedicine and Health, Chinese Academy of Sciences, Guangzhou, 510530 China; 5 Joint School of Life Science, Guangzhou Institutes of Biomedicine and Health, Chinese Academic and Sciences, Guangzhou Medical University, Guangzhou, 511436 China

**Keywords:** Kidney organoids, Mitochondria, Intermediate mesoderm progenitor like cells, Human urine cells, Partial reprogramming

## Abstract

**Background:**

The kidneys require vast amounts of mitochondria to provide ample energy to reabsorb nutrients and regulate electrolyte, fluid, and blood pressure homeostasis. The lack of the human model hinders the investigation of mitochondria homeostasis related to kidney physiology and disease.

**Results:**

Here, we report the generation of mitochondria-rich kidney organoids via partial reprogramming of human urine cells (hUCs) under the defined medium. First, we reprogrammed mitochondria-rich hUCs into expandable intermediate mesoderm progenitor like cells (U-iIMPLCs), which in turn generated nephron progenitors and formed kidney organoids in both 2D and 3D cultures. Cell fate transitions were confirmed at each stage by marker expressions at the RNA and protein levels, along with chromatin accessibility dynamics. Single cell RNA-seq revealed hUCs-induced kidney organoids (U-iKOs) consist of podocytes, tubules, and mesenchyme cells with 2D dominated with mesenchyme and 3D with tubule and enriched specific mitochondria function associated genes. Specific cell types, such as podocytes and proximal tubules, loop of Henle, and distal tubules, were readily identified. Consistent with these cell types, 3D organoids exhibited the functional and structural features of the kidney, as indicated by dextran uptake and transmission electron microscopy. These organoids can be further matured in the chick chorioallantoic membrane. Finally, cisplatin, gentamicin, and forskolin treatment led to anatomical abnormalities typical of kidney injury and altered mitochondria homeostasis respectively.

**Conclusions:**

Our study demonstrates that U-iKOs recapitulate the structural and functional characteristics of the kidneys, providing a promising model to study mitochondria-related kidney physiology and disease in a personalized manner.

**Supplementary Information:**

The online version contains supplementary material available at 10.1186/s13578-022-00909-0.

## Introduction

Chronic kidney disease (CKD) is a serious public health problem, affecting 8–16% of the global population [1, 2]. Disease progression leads to irreversible nephron loss and the effective clinical therapies remain limited. Mitochondria dysfunction is closely associated with kidney disease and underlines its roles as novel therapeutic targets [3–5]. However, owing to the lack of human experimental models, mechanisms of mitochondrial dysfunction in the development of CKD remain elusive. The generation of functional human kidney organoids may progress cell therapy development for progressive kidney disease and its corresponding models and provide a source for mechanistic studies and drug screening [6–9].

Currently, human kidney organoids can be established from pluripotent stem cells (PSCs), including embryonic stem cells (ESCs) and induced PSCs (iPSCs), and adult kidney tissues. PSCs-induced kidney organoids (P-iKOs) consist of cells from most kidney lineages, resembling an almost complete nephron in vitro [10, 11]. However, the tumorigenicity and heterogeneity of human PSCs prohibit their future clinical applications. Meanwhile, the personalized generation process of P-iKOs remains lengthy and complicated. Adult tissues derived kidney organoids, termed “tubuloids”, contain only tubular epithelial cells, and do not represent complete organs. Furthermore, the protocol for tubuloids relies on conditioned medium and expensive growth factors [12].

Thus, we wonder whether human kidney organoids can be induced in a personalized manner under defined medium in a convenient and economical way and by avoiding tumorigenicity issues. In addition, considering the vital roles of mitochondria in kidney function but lack of human models, mitochondria-rich kidney organoids are needed to allow investigation of the relationship between mitochondria and kidney physiology or disease. To develop such a system, we hypothesized that mitochondria-rich kidney organoids may be more easily induced from mitochondria-rich human urine cells (hUCs) through partial reprogramming, since it has been previously demonstrated that initially reprogrammed cells retain genetic and epigenetic memory and tend to regain their original phenotypes.

In this study, we described a robust system to generate hUCs-induced kidney organoids (U-iKOs) that contain abundant mitochondria via partially reprogramming of hUCs into intermediate mesoderm progenitor like cells (U-iIMPLCs), then in turn generate nephron progenitors and form nephron-like structures under defined medium. We found that the organoids can be generated from hUCs sourced from both healthy individuals and patients with kidney disease. The U-iKOs can recapitulate the structure and function of human nephrons physically and pathophysically in vitro. Therefore, U-iKOs may be used as an in vitro model for studying the mechanisms of kidney disease and drug screening in a personalized manner.

## Materials and methods

### Isolation and purification of urine cells

The study protocol for human urine sample collection was approved by the Ethics Committee of Nanfang Hospital, Southern Medical University, and Guangzhou Institutes of Biomedicine. Informed consent was obtained from all participants. Urine Cells were collected following the protocol described [13]. The samples of the mid-stream of urine from healthy female and male volunteers were collected and stored at 4 °C within 8 h before purification. The usual volume of specimens was 50–200 mL. The samples were centrifuged at 500 g for 8 min, and then the pellet was washed with about 10 mL DPBS supplemented with 1 × penicillin/streptomycin (lot. SH40003.01, Hyclone). After another centrifugation, the supernatant was removed and the pellets were resuspended with the urine cell culture medium supplemented with 10 μg/mL Primocin (lot. ant-pm-1, Invivogen). The urine cell culture medium was a one-to-one mixture of Renal Epithelial Growth Medium (REGM; lot. CC-3191, Lonza) and DMEM High Glucose (lot. SH30022.01, Hyclone) containing 10%FBS, 1 × L-GlutaMAX (lot. 35050-061, GIBCO), and 1 × non-essential amino acids (NEAA; lot. 11140-050, GIBCO). Then the pellets were plated in the gelatin (lot. 07903, STEMCELL) coated 6 cm culture dish and the culture medium was changed after 4 days. A few days later, hUCs grew and formed a few clusters of uniform colonies. In about 1–2 weeks, hUCs achieved confluence thereby allowing us to passage and perform reprogramming experiments.

### Reprogramming hUCs to U-iIMPLCs

hUCs were induced by oriP/EBNA episomal vectors carrying a combination of the reprogramming factors OCT4, SOX2, SV40LT, KLF4, and miR302/367 through electroporation [14, 15]. To induce U-iIMPLCs, hUCs were treated with reprogramming medium containing 5 μM CHIR99021 (lot. T2310, Targetmol), 10 ng/mL bFGF (lot. 100-18B, PeproTech), 10 ng/mL EGF (lot. AF-100–15, PeproTech), 1 μM EPZ5676 (lot. S7062, Selleck) and 10 ng/mL Activin A (lot. 120-14E-1000, PeproTech) in basic medium Advanced DMEM F12 medium (lot. 12634010, GIBCO). During this stage, check the morphology of the cells and change the fresh reprogramming medium every other day until tightly formed clusters of cells formed after 7–9 days.

### Maintaining culture of U-iIMPLCs

On the 7–9 days of reprogramming, the U-iIMPLCs clones with appropriate size were picked in the maintaining medium containing 5 μM CHIR99021, 10 ng/mL hLIF (lot. 300–05, PeproTech), and 1 μM EPZ5676 in basic medium Advanced DMEM F12 medium. The medium was changed every day. For passaging, U-iIMPLCs were dissociated with 0.25% trypsin into single cells and split at ratios of 1:3 every 2–3 days. Subcultured cells were used for growth curves, RNA extraction, and subsequent differentiation experiments.

### Differentiation of U-iIMPLCs into U-iKOs

In short, to induce posterior intermediate mesoderm cells, the U-iIMPLCs were treated with 8 μM CHIR99021, 10 ng/mL BMP4 (lot. 314-BP-01 M, R and D), and 10 ng/mL Activin A in basic differentiation medium Advanced RPMI 1640 medium (lot. 12633–012, GIBCO) supplemented with 1 × L-GlutaMAX for 1 day, followed by 10 ng/mL Activin A and 1 μM TTNPB (lot. S4627, Selleck) for 1.5 days. To induce nephron progenitor cells, the medium was changed to 1 μM CHIR99021 and 10 ng/mL FGF9 (lot. 100–23-250, PeproTech) for 1.5 days and then 10 ng/mL FGF9 with daily media change for another 3 days. To differentiate of U-iNPCs to 2D kidney organoids, the cells at day14-16 were fed with 10 ng/mL FGF9 for another 8 days and basal medium with no additional factors until harvest, with changing medium daily.

To generate 3D kidney organoids, the colonies were picked and re-aggregated into bigger cell clusters with 5–10 colonies per organoid using round bottom ultra-low attachment 96-well plates (lot. 7007-24EA, Corning) at day 14. Transfering the 3D U-iKOs onto the upper chamber of Transwell (lot. CLS3470-48EA, Corning) for air–liquid interface (ALI) culture after cell clusters aggregation for 24 h. After that, 10 ng/mL FGF9 with daily media change was performed at the bottom chamber, avoiding overflowing over the membrane until day 24.

To generate 3D kidney organoids by aggregating single cells, the picked colonies were collected and dissociated into single cells by Accutase (lot. 07920, STEMCELL). 1.5–3 × 10^5^ cells (per well) were spun down at 300 g for 5 min to aggregate into a pellet and cultured in 1 μM CHIR and 50 ng/mL FGF9 for 5 days. Then organoids were transferred to ALI culture and cultured in 10 ng/mL FGF9 until day 24 of differentiation.

### In vitro* dextran uptake assay*

Kidney organoids differentiating for at least 24 days were cultured with 100 μg/mL fluorescence-labeled 10 kDa (lot. D22914, Thermo Fisher Scientific), 70 kDa (lot. D1818, Thermo Fisher Scientific), and 2000 kDa (lot. D7139, Thermo Fisher Scientific) dextran for 4 h. Then, kidney organoids were cultured in the basic medium without dextran for another 24 h and then were fixed for immunofluorescence analysis. The medium was only added into the bottom chamber of transwells.

### Nephrotoxicity assay with cisplatin and gentamincin

Kidney organoids differentiating for at least 24 days were treated with the basic differentiation medium supplemented with 5 μM cisplatin (lot. P4394, Sigma), 5 mg/mL gentamicin, or sterile water as a negative control for 24 h, after which 3D U-iKOs were harvested for immunostaining and gene expression analysis.

### Cyst induction

To initiate cytogenesis, kidney organoids at D24 were treated with basal culture medium supplemented with either 5 μM forskolin (FSK; lot. C10901, ChemBest) or sterile water as a negative control for 4 days, after which 3D U-iKOs were harvested for immunostaining and gene expression analysis.

### Implantation of hUCs derived kidney organoids onto chick chorioallantoic membrane (CAM).

Briefly, fertilized chicken eggs were obtained from a commercial supplier in Qingyuan City (in Guangdong, China). Eggs were incubated in a humidified atmosphere in a 37.5 °C incubator. At embryonic day (ED) 3.5, 2 mL of albumin was extracted from the egg using a 21-gauge syringe. Then a small window was opened by cutting the eggshell and was sealed with a breathable membrane. At ED 8–9, aggregated U-iNPCs at day 15–17 were implanted onto the surface of the CAM and incubated for 7 additional days.

### Histological analysis

Grafts of CAM and 3D U-iKOs were fixed in 4% paraformaldehyde at 4 °C overnight and 3D U-iKOs for 1 h at room temperature (RT). Then samples were transferred to sucrose gradient (15%-30% sucrose, w/v) treatment overnight and embedded in OCT compound (Leica). For histological analysis, 10 μm thick sections generated using a cryostat (Leica) were stained with Hematoxylin and Eosin (H&E). Images were captured using Pannoramic MIDI and processed by CaseViewer (3DHISTECH).

### Immunostaining and confocal imaging

In short, fixed cells or slides were permeabilized by 0.2% Triton X-100 for 15 min and incubated in blocking buffer (PBS supplemented with 5% FBS, 2.5% BSA) for 1 h at RT. Subsequently, samples were washed, incubated with primary antibodies overnight at 4 °C, and incubated with Alexa-Fluor secondary antibodies at RT for 1 h. At last, nuclei were counterstained with DAPI (lot. D9542, Sigma) for 1 min at RT. Immunofluorescence was visualized using LSM800 (Carl Zeiss). The antibodies and associated information are provided (Additional file 2: Table S1).

### Transmission electron microscopy (TEM)

In brief, kidney organoids were fixed in 2.5% glutaraldehyde and 2% paraformaldehyde overnight at 4 °C and washed with 0.1 M phosphate buffer 6 × 15 min. Then, samples were stained with 1% osmium tetroxide solution for 1 h, followed by 6 × 15 min washes. Subsequently, samples were dehydrated in ethanol gradient (50%, 70%, 80%, 90%, 100%, and 100%) and acetone (100%) before infiltration with acetone: epon resin mixture (1:1) for 2 h and then absolute resin overnight. The samples were reduced in size by cutting into small blocks (about 2 × 2 mm) and then subjected to ultra-thin sections. Images were collected using either Tecnai G2 Spirit TEM (FEI).

### Quantitative RT-PCR (qRT-PCR)

Total RNA was isolated using RNA-easy Isolation Reagent (lot. R701-02, Vazyme) and 1 μg RNA was reverse transcribed using HiScript II Q RT SuperMix for qPCR (lot. R222-01, Vazyme). Then, qRT-PCR was carried out using ChamQ SYBR qPCR Master Mix (lot. Q311-03, Vazyme) and CFX Connect PCR machine (Bio-Rad). GAPDH was used as the housekeeping gene. Values were calculated by the delta CT method and all data were analyzed in triplicates. All primer sequences for the human sample are listed (Additional file 2: Table S2). Two-tailed Student’s t-test was used to determine statistical significance between the control and test groups. The analysis was performed using Graphpad Prism.

### Western blot analysis

Whole-cell extracts of cell samples were subjected to 10–12% SDS-PAGE and transferred to PVDF membranes (lot. ISEQ00010, Millipore). Then, these PVDF membranes were incubated with primary antibodies at 4 °C overnight. After being washed 3 × 10 min in TBST (TBS supplemented with 0.1% Tween-20), the membranes were incubated with HRP-conjugated secondary antibodies (Beyotime) for 1 h at RT, followed by 3 × 10 min washes. Then the membranes were visualized with a GelView 6000 Plus (BLT Photon Technology). The antibodies and associated information are provided  (Additional file 2: Table S1).

### Bulk RNA-seq library construction

After the total RNA extracts were prepared, RNA libraries were established using VAHTS^®^ Universal V8 RNA-seq Library Prep Kit for Illumina (lot. NR605-02, Vazyme) according to the manufacturer’s protocol. In brief, 0.5–1 μg of total RNA was used for library construction. mRNA was enriched by the Poly(A) method with VAHTS mRNA Capture Beads (lot. NR1-01, Vazyme), followed by cDNA library preparation, library quality control, and sequencing using NovaSeq 6000 (Illumina).

### ATAC-seq library construction

ATAC-seq was performed using TruePrepTM DNA Library Prep Kit V2 for Illumina^®^ (lot. TD501-01, Vazyme), as previously described [16]. In brief, pellets of a total of 5–8 × 10^4^ cells were re-suspended in 50μL lysis buffer. Then the suspension was subjected to transposition reaction and purification with MinElute Kit (QIAGEN). After PCR amplification of transposed DNA fragments, the amplified libraries were purified using a Qiaquick PCR (QIAGEN) column. Then, purified libraries were assessed by gel electrophoresis and Qsep100 (Bioptic). Finally, the ATAC library was sequenced on NovaSeq 6000 (Illumina).

### Single cell RNA-seq (ScRNA-seq) library preparation

Kidney organoids generated by colonies aggregation for at least 24 days were dissociated into single cells with Accutase at 37 °C and filtered through a 40 μm filter thrice to remove cell clumps. A minimum of 1 × 10^4^ cells were loaded per well for single-cell RNA-seq library preparation with the Chromium Next GEM Single Cell 3’ Reagent Kits v3.1 (lot. CG000316, 10 × Genomics) according to the manufacturer’s recommendations. Libraries generated were sequenced on Illumina NovaSeq 6000 platform.

### ATAC-seq data analysis

All of the sequencing data were aligned to the human genome assembly (hg38) using bowtie2 (v.2.2.5) with the following options (− p 10–very-sensitive–end-to-end–no-unal) [17]. Duplicate reads were discarded using samtools and picard tools. For visualization, the aligned BAM files were converted to read coverage files (bigWig format) using deepTools and signals were normalized to RPKM value [18]. Then, MACS2 was used to call narrow peaks with default options [19]. After a series of threshold filtering, we set 16 as the threshold value to define open/closed regions. If the signal RPKM of the sample is lower than this value, it is marked as ‘closed’, otherwise, it is annotated as ‘open’. Motif analysis was performed using the findMotifsGenome.pl program in HOMER (v.4.10) with options (− p4-size given) [20]. Peaks annotation was performed using ChIPseeker (v1.22.1). ChIPpeakAnno was used to plot Venn diagrams of peaks. Other analyses were performed using glbase with personalized scripts [21].

### Bulk RNA-seq data analysis

The paired-end sequenced reads were aligned to a transcriptome index generated from the GENCODE annotations transcriptome (Hg38), using RSEM, bowtie2 [22]. And the Transcripts Per Kilobase Million (TPM) values calculated were used for downstream differential expression analysis based on the R/Bioconductor (v3.6.1). We calculated the top 5000 highly variable genes and then used them for principal component analysis (PCA) and correlation analysis. A fold change of 1.5 was used as the threshold to filter the differentially expressed genes. Gene ontology (GO) analysis was performed using the R package clusterProfiler [23]. The associated plots were generated using the ggplot2 package (v3.3.2).

### ScRNA-seq analysis

Data quality was assessed with Fastp. Paired-end sequencing reads were aligned to hg38 genome using the STAR (v2.7.6a) with parameter (–soloType Droplet–soloFeatures Gene–soloCBstart 1–soloCBlen 16–soloUMIstart 17–soloUMIlen 12) [24]. The read1 data contains the first 16 bp of barcode sequence, and the next 12 bp were recorded as unique molecular identifiers (UMIs). The read2 with 91 bp was used for genome alignment. We used the Python library Scanpy (v1.6.0) and R library Seurat (v3.1.4) to pre-process the data [25, 26]. We filtered low-quality cells with less than 1,500 and greater than 10,000 detected genes, the sum of UMI counts greater than 60,000, and the percentage of mitochondrial genes more than 0.2. And then we predicted doublets and removed 44 cells with parameter (threshold = 0.4) using scrublet, a single-cell remover of doublets. This pro-processing step results in 10,739 cells with 4591 median genes from 3D U-iKOs, and 10,125 cells with 2815 median genes from 2D U-iKOs samples. We used default settings in the Scanpy to normalize the gene expression counts with the function ‘pp.normalize_per_cell’ and ‘pp.log1p’, and identified the top 4,000 highly variable genes (HVGs) with the function ‘pp.highly_variable_genes’. We scaled data using the function ‘pp.scale’ with all detected genes. The HVGs were conducted for PCA and then found the neighbors for each cell with 30 PCs. UMAP was used for dimensionality reduction and visualization in 2D space with function ‘tl.umap’. Leiden clustering was processed on the HVGs calculated across each sample, with function ‘tl.leiden’ and parameter (resolution = 0.5).

We then used the Seurat function ‘FindAllMarkers’ to calculate the differentially expressed genes for each cluster. Cluster identity was performed by comparing these genes with a list of well-known genes for kidney cell types provided in Fig. 3B. And subclustering was performed described above. And the GO analysis was performed using the R package clusterProfiler.

### Integrated analysis of multiple datasets

To compare the cell types derived from different organoid protocols and fetal/adult human kidney tissue, we collected iPSC-derived organoids T-iKOs, M-iKOs and C-iKOs, and human fetal kidney at week16 [27–29]. We re-analyzd these data and identified cluster with the information provided in these articles. We then performed comparative analysis on multiple datasets using Seurat V3. Firstly, it selected 2000 features that are repeatedly variable across the multiple datasets and then integrated into a combined data using function ‘IntegrateData’. The canonical correlation analysis (CCA) was performed to identify common sources of variation across the datasets.

We calculated the differential expression genes using the function ‘FindAllMarkers’ with the for each data, and dotplots showed selected genes were significantly different.

## Results

### U-iKOs generation under a defined medium

**Fig. 1:**
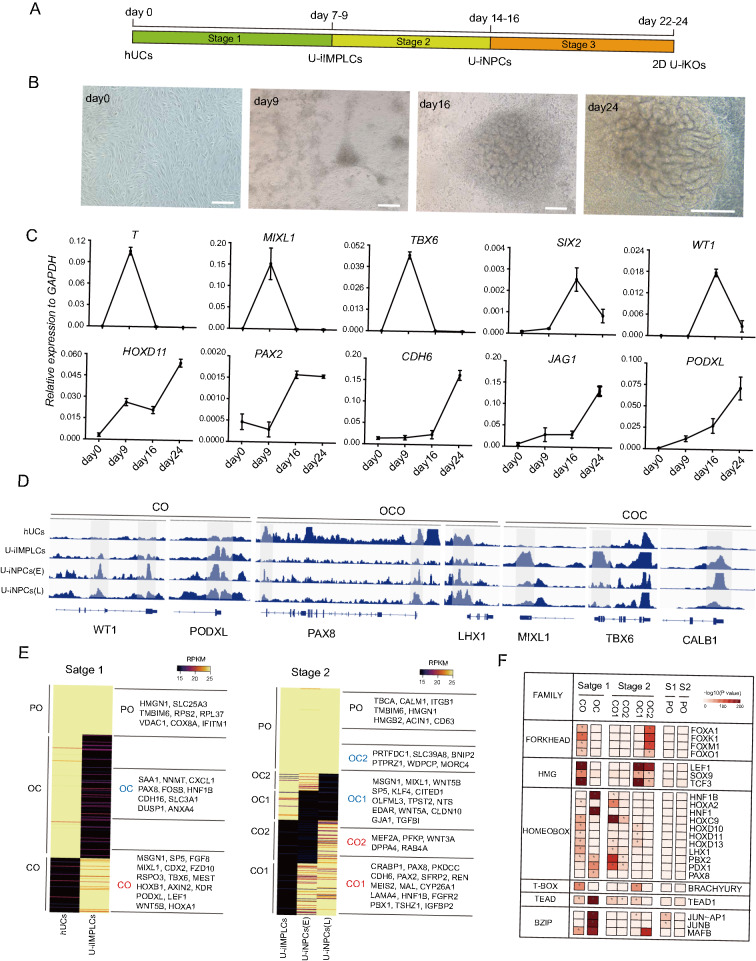
Generation of 2D kidney organoids from hUCs. **A** Schematic illustration of 2D kidney organoids starting from hUCs (human urine cells), hUCs-induced intermediate mesoderm progenitor like cells (U-iIMPLCs), hUCs-induced nephron progenitor cells (U-iNPCs), hUCs-induced kidney organoid (U-iKOs) at the indicated time points. **B** Representative bright-field images during A at day0, day9, day16, and day24. Scale bars, 250 μm. **C** Gene expression analysis for markers of the primitive streak (T, MIXL1, and TBX6), nephrogenic intermediate mesoderm (WT1 and HOXD11), nephron progenitor (SIX2, WT1, and PAX2), podocytes (PODXL and WT1), tubule epithelial cells (CDH6 and JAG1) during differentiation. The relative expression of each transcript to GAPDH expression is presented as the mean ± SEM (n = 6). **D** Representative loci for the close-to-open (CO), open-to-close-to-open (OCO), and close-to-open-to-close, (COC) peaks at different stages at hUC (day0), U-iIMPLCs (stage1, day9), early U-iNPCs (stage2, day16) and late U-iNPCs (stage2, day19). **E** Dynamic CO, OC, and permanently open (PO) chromatin regions are clustered for hUC, U-iIMPLCs, U-iNPCs (E) and U-iNPCs (L). Representative genes are noted for each subgroup on the right side. **F** Heatmaps for the motif enrichment among the OC, CO and PO subgroup peaks for stage1, and stage2 as shown in E. *p < 1e-30

Previously, we demonstrated that hUCs are reprogrammable into iPSCs, neural progenitor cells (NPCs) and cardiomyocytes by defined factors [13, 30, 31], indicating the high cell fate plasticity of hUCs. Hence, we speculated whether hUCs could be reprogrammed into other cell fate states, such as kidney lineage-associated progenitor cells, which are the developmental ancestors of differentiated hUCs. To test this hypothesis, we isolated and cultured hUCs in renal epithelial growth medium (REGM), and nearly 100% of hUCs expressed the tubular epithelial cell marker, PAX8. Furthermore, hUCs were reprogrammed in a series of defined medium. Through screening and optimization, we eventually developed a defined medium including the following chemicals: CHIR99021 (5 μM), EPZ5676 (1 μM) and Actinvin A (10 ng/mL), bFGF (10 ng/mL), and EGF (10 ng/mL) to convert hUCs into U-iIMPLCs within 9 days. Followed by step-wise differentiation under the defined medium, U-iIMPLCs were induced into nephron progenitor cells (U-iNPCs) at 14–16 days and then U-iKOs at 22–24 days (Fig. 1A, B). These intermediate identities were confirmed through gene expression analysis such as T, MIXL1, and TBX6 for primitive streak; WT1 and HOXD11 for nephrogenic intermediate mesoderm; SIX2, WT1, and PAX2 for nephron progenitors; Podocalyxin (PODXL) and WT1 for podocytes, and CDH6 and JAG1 for tubule epithelial cells (Fig. 1C, Additional file 1: Fig. S1A, F). Furthermore, they did not express markers for the pluripotent, definitive endoderm, or ectoderm (Additional file 1: Fig. S1B). We successfully generated organoids from 11 individuals, including 8 healthy volunteers and 3 patients with CKD from both males and females (Additional file 1: Fig. S1C). Patient-derived urine cells underwent a similar process of cellular morphological changes and gene expression profile changes to healthy volunteers (Additional file 1: Fig. S1D, E).

To further characterize the process of U-iKOs generation, we profiled the chromatin accessibility and transcriptome dynamics using ATAC-seq and RNA-seq respectively at each stage. At the chromatin level, ATAC-seq analysis revealed that genomic loci associated with MIXL1, TBX6, WT1, PAX8, LHX1, CALB1, and PODXL underwent closing or opening as shown in Fig. 1D. Notably, the PAX8 locus closed at the first stage, but then opened upon acquisition of kidney lineage fate (Fig. 1D), whereas the TBX6 locus opened at the first stage but then closed at stage 2 (Fig. 1D). Globally, loci underwent dynamic close-to-open (CO), or open-to-close (OC) remodeling during the entire process as shown in Fig. 1E. At each transition, we identified critical genes up- or down-regulated by time-course RNA-seq (Additional file 1: Fig. S2A), with corresponding biological pathways including mesoderm development, kidney development, and epithelial cell morphogenesis involved (Additional file 1: Fig. S2B–D). Interestingly, loci with CO or OC were enriched with specific transcription factors, such as those from the FORKHEAD, HMG, HOMEBOX, T-BOX, TEAD, and BZIP families (Fig. 1F), reflecting the specific roles of each factor during the two main transitions from U-iIMPLCs to U-iNPCs and then from U-iNPCs further to U-iKOs.

**Fig. 2:**
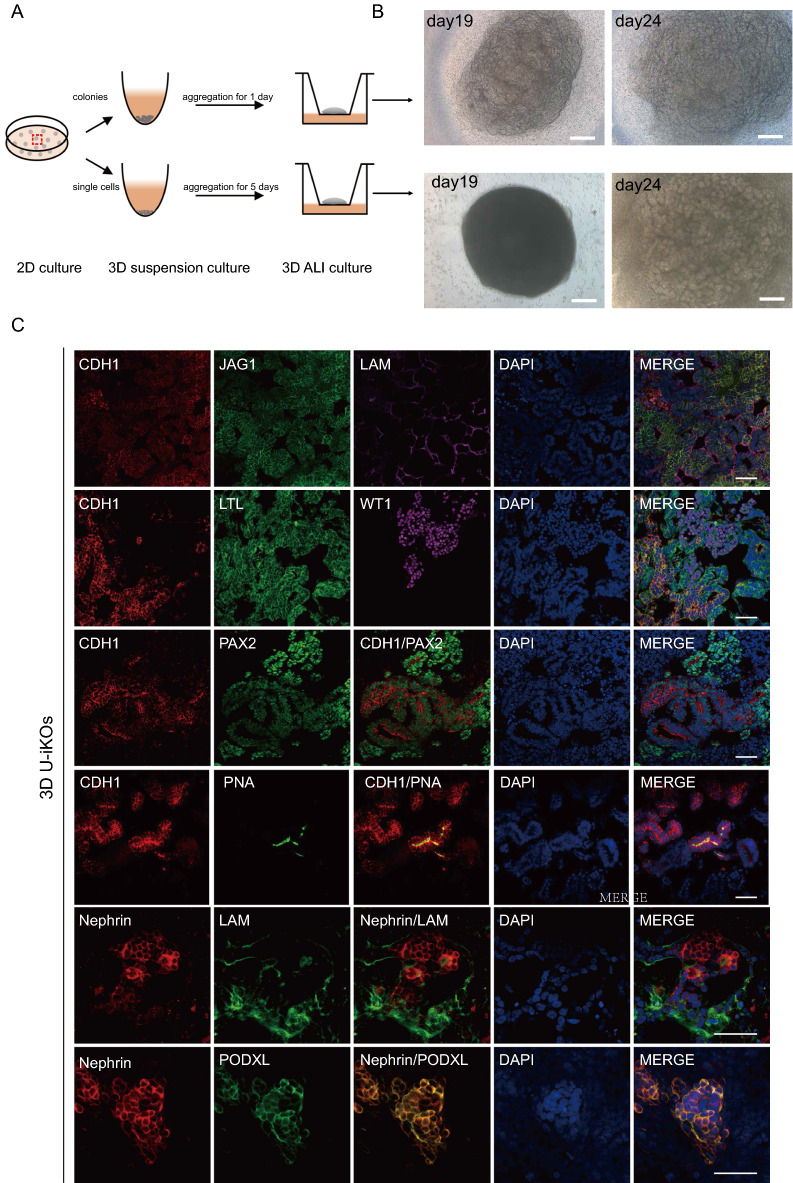
Establishment of 3D U-iKOs. **A** Schematic illustration of 3D U-iKOs. Colonies at day14 in Fig. 1 were picked and then aggregated as pellets or single cells in suspension and air–liquid interface (ALI) culture to form 3D U-iKOs at indicated time points in B. **B** Representative bright-field images of 3D U-iKOs as in A. Scale bars, 250 μm. **C** Immunostaining analysis of nephron segment-specific markers for podocytes (PODXL, nephrin, and WT1), proximal tubule (LTL), medial tubule (JAG1), distal tubule (PAX2, PNA and CDH1), and basement membrane (LAM). Scale bars, 50 μm

The surprisingly simple way to generate organoids directly further encouraged us to design methods to generate 3D organoids. As shown in Fig. 2A, we came up with a relatively straightforward procedure to generate 3D in 24 days. As shown in Fig. 1, we picked colonies from a 16-day culture and then either aggregated them directly for 1 day or dissociated them into single cells before aggregation for 5 days. These aggregated cells were then placed in the 3D air–liquid interface (ALI) culture, as illustrated in Fig. 2A. On day 19, cells from colonies grew into organoids, whereas those from single cells remained as spherical cysts (Fig. 2B). Yet, at day 24, both procedures generated organoids with specialized and complex structures (Fig. 2B, right panels). We further characterized these organoids by performing immunostaining for the specific markers as shown in Fig. 2C. We accurately identified kidney-specific structures and cells such as podocytes (PODXL, nephrin, and WT1), proximal tubules (LTL), medial tubules (JAG1), distal tubules (PAX2, PNA and CDH1), and basement membrane (LAM). These results suggested that high-quality 3D organoids can be generated using aggregation and ALI culture conditions. Together, through partial reprogramming and staged differentiation under defined medium, we obtained personalized kidney organoids within a month. To obtain kidney organoids from PSCs in a personalized way, somatic cell reprogramming is required to obtain iPSCs and then proceed to the differentiation process, which typically takes 2–3 months following literature (Fig. 1G) [6, 32].

### Long-term expanding U-iIMPLCs for U-iKOs generation

**Fig. 3:**
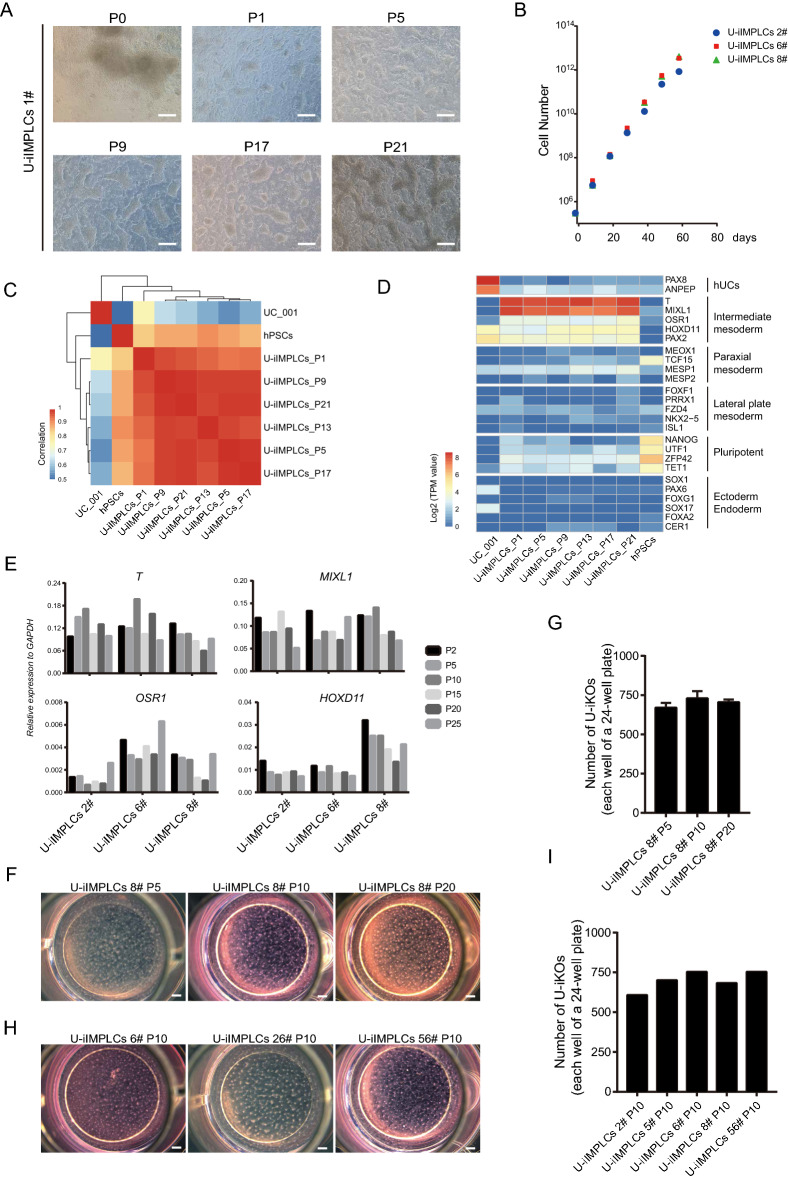
Efficient maintained U-iIMPLCs retain nephrogenic potential. **A** Cell morphology of U-iIMPLCs subcultured at different passages. The same strain of U-iIMPLCs 1# with different passages P0, P1, P5, P9, P17, and P21 are presented. Scale bars, 250 μm. **B** Growth curve of U-iIMPLCs for different strains of U-iIMPLCs 2#, 6#, and 8#. **C** Correlation analysis showing gene expression profiles of hUCs, hPSCs, and U-iIMPLCs 1# maintained at different passages. **D** Gene expression analysis for hUCs, intermediate mesoderm, paraxial mesoderm, Lateral plate mesoderm, pluripotent, ectoderm, and endoderm markers of the cells shown in C. **E** Gene expression analysis for intermediate mesoderm progenitor markers for different strains with different generations. The relative expression of each transcript to GAPDH expression is presented as the mean ± SEM. **F** Representative stereo microscope images showing 2D kidney organoids differentiated from the same strain of 8# U-iIMPLCs at different passages in a single well of a 24-well plate. Scale bars, 500 μm. **G** The number of 2D U-iKOs in F. **H** Representative stereo microscope images showing 2D kidney organoids differentiated from different strains at passage 10. Scale bars, 500 μm. **I** The number of 2D kidney organoids is shown in H

During daily cell culture, certain hUCs from some individuals or batches proliferated slowly or poorly, which lengthened the time required to collect sufficient hUCs for reprogramming. Therefore, we examined whether any progenitor state during the U-iKOs generation could expand long-term in vitro, which is of vital importance for optimization, functional validation, and future application. By optimizing culture conditions, we succeeded in developing a defined medium including CHIR99021 (5 uM), EPZ5676 (1 μM), and hLIF (10 ng/mL) to support long-term growth and expansion of U-iIMPLCs, but not for U-iNPCs or U-iKOs (data not shown). Under the defined medium, U-iIMPLCs grew in tight clusters and expanded in vitro for more than 20 passages within 80 days (Fig. 3A, B). RNA-seq indicated that the stable transcription profiles of U-iIMPLCs were distinct from those of hUCs and hPSCs (Fig. 3C). In detail, U-iIMPLCs expressed intermediate mesoderm specific markers OSR1 and HOXD11 but not the paraxial mesoderm markers MSGN and MESP1, lateral mesoderm markers FOXF1 and NKX2.5, pluripotent genes NANOG, ectodermal genes SOX1 and PAX6, and endodermal genes SOX17 and FOXA2 (Fig. 3D). Consistently, qRT-PCR analysis showed that U-iIMPLCs from three different cell lines at passages 2, 5, 10,15, 20, and 25 similarly expressed T, MIXL1, OSR1, and HOXD11 similarly (Fig. 3E). To assess the potential of U-iIMPLCs for U-iKOs generation, we chose U-iIMPLCs from the same cell line at passage 5, 10, 15 and 6 different cell lines at passage 10 and seeded at 10‒12.5 × 10^4^ cells/cm^2^ for U-iKOs induction (Fig. 3F, H). Following a two-stage process, U-iIMPLCs efficiently differentiated into kidney lineages and formed more than 600 2D U-iKOs per well of 24-well cell culture plate (Fig. 3G–I). Taken together, we concluded that U-iIMPLCs can self-renew in vitro under defined medium and possessed the potential to generate U-iKOs efficiently.

### Characterizing U-iKOs by single cell RNA sequencing (scRNA-seq)

**Fig. 4:**
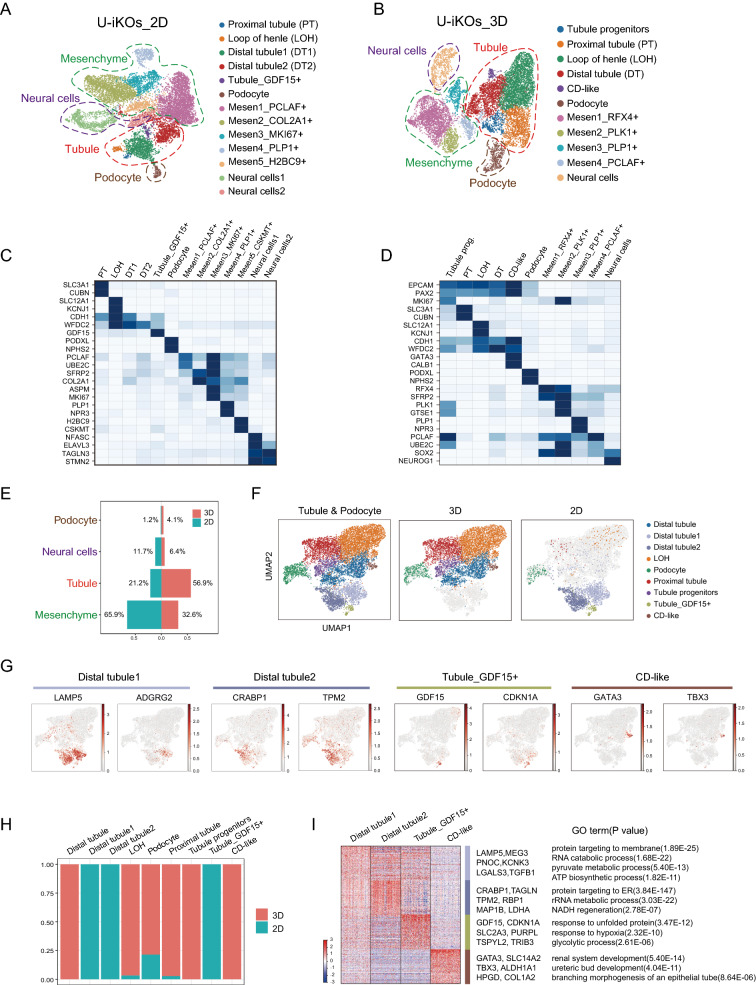
ScRNA-seq profiling of cell types in U-iKOs. **A** UMAP projection of 10,125 single cells from 2D U-iKOs into 13 distinct clusters. Four main cell types are circled on the diagram with different colors. **B** UMAP projection of 10,739 single cells from 3D U-iKOs into 11 distinct clusters. Four main cell types are identified in A. **C** Heatmap of well-known markers and signature genes in A. **D** Heatmap of well-known markers and signature genes in B. **E** Barplot of the tubule, podocyte, neuron, and mesenchyme between 3 and 2D U-iKOs. **F** UMAP projection of tubule and podocyte from 3 and 2D U-iKOs into 9 clusters. **G** Signature genes in distal tubule1, distal tubule2, tubule_GDF15 + , and collecting duct (CD). **H** Barplot of 3D and 2D cells in each cluster in F. **I** Heatmap of differentially expressed genes between 4 clusters in F, selected signature genes, and GO term with p values in the right

Cell type diversities in organoids were further described using scRNA-seq. These results showed that the 10,125 single cells from 2D U-iKOs could be classified into 13 distinct clusters (Fig. 4A), while the 10,739 single cells from 3D U-iKOs into 11 distinct clusters (Fig. 4B) with high quality (Additional file 1: Fig. S3A). Overall, four major cell types were identified, including podocytes, tubules, mesenchyme, and neural cells, which were consistent with the immunostaining results described above. These main types were further classified into 13 and 11 subtypes in the 2D and 3D organoids respectively with specific markers identified (Fig. 4C, D). The heatmap shows similar cell types in 2D and 3D organoids with a high correlation (Additional file 1: Fig S3B). For the 2D culture of U-iKOs, the predominant cell types were mesenchyme related with five distinct subtypes, specifically labeled with PCLAF/UBE2C (subtype1), SFRP2/COL2A1 (subtype 2), PLP1/NPR3 (subtype 4) and H2BC9 and CSKMT (subtype 5) (Fig. 4C). Interestingly, subtype 3 appeared to share markers with subtypes 1 and 2, suggesting that these cells may be intermediates (Fig. 4C). On the other hand, 3D U-iKOs had ~ 57% tubular epithelial cells, suggesting that the 3D condition favors the tubular structure. Reflecting being more mature, 3D U-iKOs featured a collecting duct-like that was absent from the 2D ones (Fig. 4B, D). Furthermore, the various cell types in 3D U-iKOs had more complex markers than their counterparts in 2D, again reflecting a more mature state (Fig. 4D). The difference between 2 and 3D became apparent when cell type diversities were compared directly, as shown in Fig. 4E, F, as 3D organoids were enriched with the loop of Henle, proximal tubule, distal tubule, podocyte, and tubule progenitors, and collecting tubule compared to 2D organoids with podocyte and distal tubule. It was of interest to note that, even with the same origin of cells and the same culture media, culture dimensions, i.e., 2D vs. 3D, they conferred drastically different cell fates (Fig. 4G–I). For instance, 2D generated more distal tubule cells and tubule GDF15 + cells (Fig. 4G–I). Collecting tubule-like cells were unique in only 3D conditions, reflecting the spatial requirement for specific cell types such as collecting tubule lineage (Fig. 3G–I). Hence, our findings demonstrated that 3D is superior to 2D in generating more mature kidney organoids.

The podocytes were further classified into four different groups, including NPHS2, SUSD3, MKI67, and CLDN6 respectively (Additional file 1: Fig. S3C). These four groups of cells appeared to capture different stages of podocyte development. The NPHS2 + cells are the mature type expressing PODXL, PTPRO, CLIC5, and other genes critical to glomerular structure and function (Additional file 1: Fig. S3C, D, blue). Marked by CLDN6, a tightly clustered group of podocytes (Additional file 1: Fig. S3C, red), expressed genes critical for kidney morphogenesis, nephron development, and epithelium development (Additional file 1: Fig. S3D, bottom). The MKI67 + cells actively proliferated and expressed genes involved in DNA replication, nuclear division, and chromosomal segregation (Additional file 1: Fig. S3C, green). The SUSD3 + cells expressed genes actively involved in metabolic processes and hypoxia response (Additional file 1: Fig. S3D).

The proximal tubule cluster of cells were subdivided into 5 groups, marked by IRX1, CUBN, WT1, SLC2A1, and MKI67 respectively (Additional file 1: Fig. S3E). Remarkably, CUBN + proximal tubule cells reflected the stage of differentiation and also expressed genes related to reabsorption function, such as LRP2, APOM, and DAB. Again, the MKI67 marked the proliferation group that also expressed genes implicated in DNA replication and the cell cycle (Additional file 1: Fig. S3F). The other three groups of cells reflected various stages of development, as exemplified by the mature cell types in the proximal tubule /loop of Henle (Additional file 1: Fig. S3E, F). The proximal tubule is a major kidney function structure and the recapitulation of these cell types further demonstrated the potential of our approach. The loop of Henle and the distal tubule was well represented well in the organoids, including diverse cell types from GATA3 + cells to SLC12A1 + cells, again reflecting the fates of cells undergoing differentiation and maturation (Additional file 1: Fig. S3G, H). Mature cell types marked by SLC12A1, CLDN10, ATP1A2, and other genes depict the function of the loop of Henle with ion homeostasis and regulation of pH. The GATA3 + showed the expression of collecting duct-specific genes SLC14A2, TBX3, and SLC12A2, which produced progenies with branching morphogenesis of an epithelial tube and ureteric bud morphogenesis. (Additional file 1: Fig. S3G, H).

Together, the identification of individual cell types within the organoids suggested that the 3D condition recapitulated various aspects of in vivo development and generated cell types that are available under physiological condition.

### U-iKOs are rich in mitochondria

**Fig. 5:**
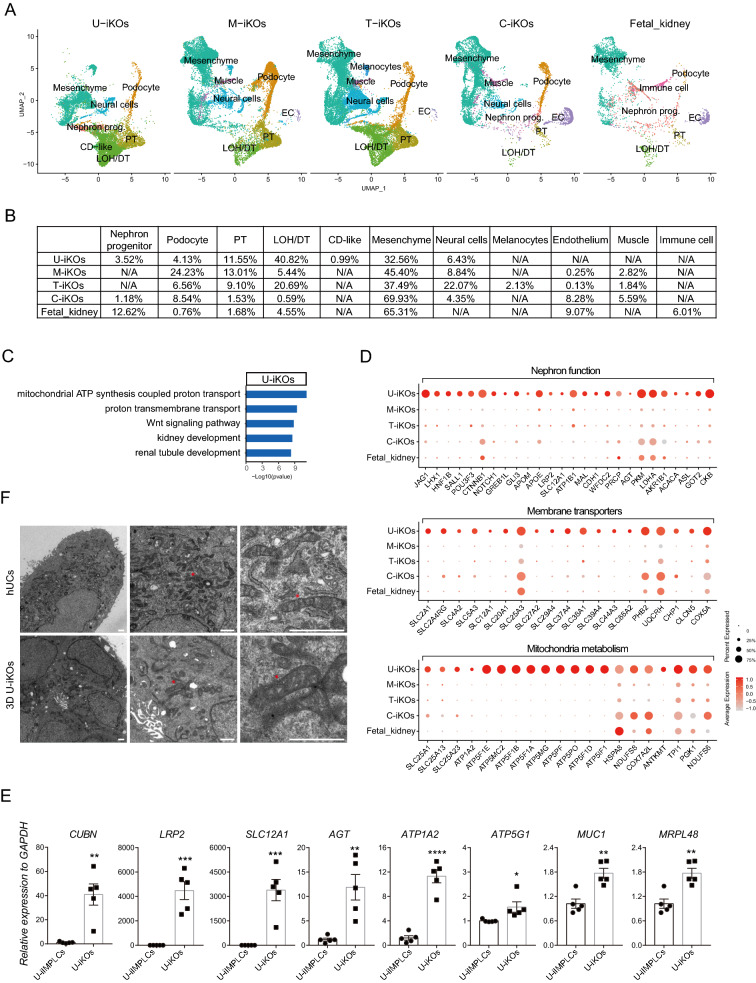
U-iKOs are rich in mitochondria. **A** The UMAP shows the integration of 3D U-iKOs, M-iPSC, T-iPSC, C-iKOs, and human fetal kidney, with cell types on the right. M-iKOs, iPSC-induced organoids with protocol by Morizane; T-iKOs, iPSC-induced organoids with protocol by Takasato; C-iKOs, iPSC-induced organoids from Combes’s study; Fetal_kidney, human fetal week16 kidney from Lindström’s study. **B** Comparison of the proportions of cell types in 3D U-iKOs, M-iKOs, T-iKOs, C-iKOs and human fetal kidney. **C** GO analysis of differentially expressed genes in 3D U-iKOs. **D** Dotplot shows differentially expressed genes of 3D U-iKOs related to nephron function (top panel), membrane transporters (middle panel), and mitochondia metabolism (bottom panel) compared with with other data. **E** Gene expression analysis for differentially expressed genes in U-iKOs in D of the mature tubule (CUBN, LRP2, and SLC12A1), AGT (angiotensinogen), and mitochondria metabolism (ATP1A2, ATP5G1, MUC1, and MRPL48). The relative expression of each transcript to GAPDH expression is presented as the mean ± SEM (n = 5). *P < 0.05 vs. the U-iIMPLCs; **P < 0.01 vs. the U-iIMPLCs; ***P < 0.001 vs. the U-iIMPLCs; ****P < 0.0001 vs. the U-iIMPLCs. (F) TEM of hUCs (upper panel) and 3D U-iKOs (botttom panel). Abundant mitochondria indicated with red asterisks. Scale bars, 1 μm

Due to the unique way of U-iKOs generation, we hypothesized that U-iKOs might possess some different features from the developed P-iKOs recently. In addition, shortage of the research on the similarity between organoids and human fetal kidney also inspired us to make comparisons. To address this, we collected similar scRNA-seq datasets generated from P-iKOs and published human fetal kidney at 16 weeks, and then made comparisons, showing that U-iKOs and P-iKOs shared most tubule epithelial cells and mesenchyme cells (Fig. 5A). In detail, U-iKOs contained collecting duct-like cells which were not generated in P-iKOs, and a higher percentage of the loop of Henle/distal tubule (Fig. 5B). In contrast, we verified that P-iKOs and fetal kidney contained the endothelial cells subpopulation that was absent in U-iKOs. Off-target cells, such as muscle cells and melanocytes, were generated in P-iKOs but not in U-iKOs (Fig. 5B), suggesting cell fate commitment of U-iIMPLCs for U-iKOs induction. The differences in cellular composition between U-iKOs, P-iKOs and fetal kidney inspired us to make an in-depth analysis in molecular level. GO term analysis revealed that U-iKOs were specifically enriched in mitochondria ATP synthesis and metabolic process, proton transmembrane transport, and kidney development (Fig. 5C), whereas differentially expressed genes in P-iKOs were associated with Golgi vesicle transport, RNA splicing, and cytoplasmic translation (Additional file 1: Fig. S4A–D). Compared to organoids, human fetal kidney was enriched in response to unfolded protein and response to temperature stimulus with higher expression level of HSPA1A, HBG2 and GPC3, consistent with those previously described [28]. Consistently, we found that U-iKOs expressed a higher level of kidney function associated genes, such as LRP2 (megalin; macromolecules and numerous ligands reuptake), CUBN (endocytic receptor), AGT (pre-angiotensinogen), PRCP (a serine exopeptidase that acts on angiotensin II), PKM (glycolysis), and LDHA (oxidoreductase) (Fig. 5D). The results revealed that membrane transporters and mitochondria metabolism-related genes were specifically expressed in U-iKOs but not in P-iKOs, such as SLC12A1 (sodium, potassium, and chloride ion cotransporter), SLC5A3 (sodium transport), SLC25A1 (tricarboxylate transporter), SLC25A3 (phosphate carrier protein, mitochondrial), and ATP1A2 (sodium/potassium-transporting ATPase) (Fig. 5D), suggesting that cells in U-iKOs may contain abundant mitochondria. The expression of these genes was also validated by qRT-PCR detection (Fig. 5E). Nephron function maker, such as SLC12A1, LRP2 were validated in 3D U-iKOs (Additional file 1: Fig. S4E). To further confirm these results, we examined cellular organelles in hUCs and U-iKOs using TEM and showed that both proximal and distal tubule epithelial cells consisted of a number of mitochondria in the cytoplasm (Fig. 5F). Taken together, these results demonstrated that U-iKOs are rich in mitochondria, validating our hypothesis that mitochondria-rich kidney organoids may be easily induced from mitochondria-rich human urine cells through partial reprogramming.

### Structure and function of developed 3D U-iKOs

**Fig. 6:**
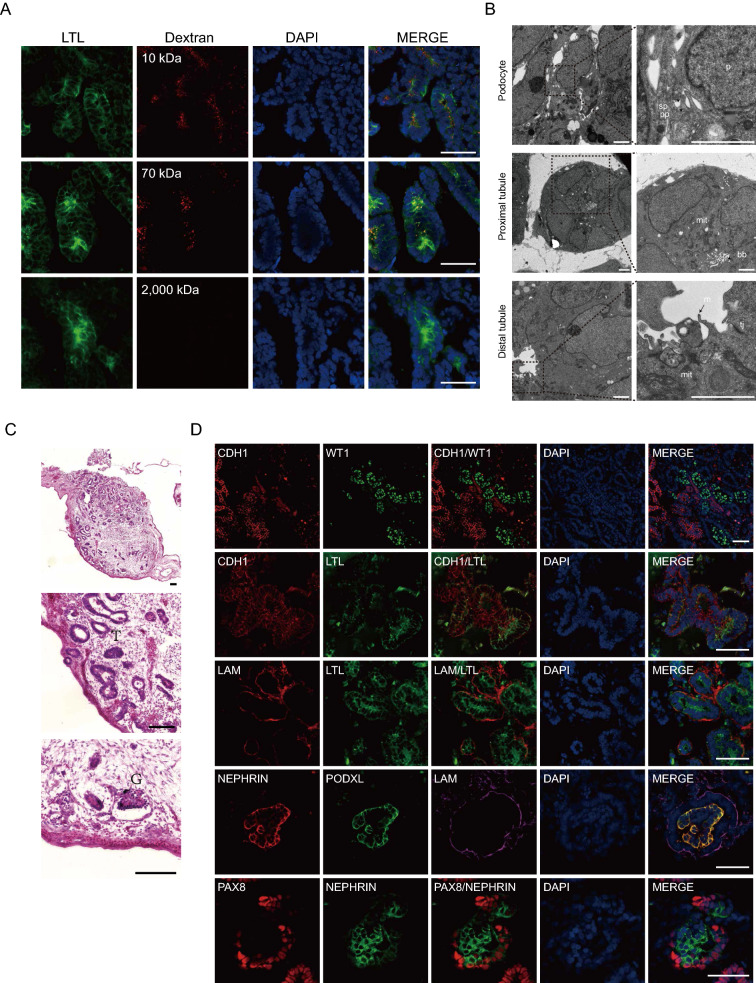
Structure and function of 3D U-iKOs. **A** Immunofluorescence analysis of fluorescence-labeled dextran uptake assay in 3D U-iKOs for 10 kDa, 70 kDa, and 2000 kDa of dextran. Scale bars, 50 μm. **B** TEM of 3D U-iKOs (day 24). Podocyte (p) with characteristic large nuclei and primary processes (pp) and secondary processes (sp) in the upper panel. A putative proximal tubule cells with enriched mitochondria (mit) and a lumen filled with closely packed microvilli and the brush border (bb) in the middle panel. A putative distal tubule with relatively sparse short microvilli (m) and tight junctions (tj) in the bottom panel. Scale bars, 2 μm. **C** H and E staining of implanted kidney organoids in the CAM for 6 days. Magnified views of glomerular (G) and tubular (T) cells are shown. Scale bars, 100 μm. **D** Confocal microscopy images of implanted organoids in C stained for parietal epithelial cells maker (PAX8), bowman’s capsule basement membrane (LAM), and tubular markers (LTL, CDH1, and PAX8). Scale bars, 50 μm

ScRNA-seq provided evidence that the 3D organoids possessed the cellular diversity to perform kidney functions. To further characterize the structure and anatomy of the organoids, we observed the uptake of dextran under 100 kDa and the exclusion of those above 2000 kDa (Fig. 6A). The results showed that these organoids function similarly to the kidneys. Additionally, we showed that the ultrastructure of the organoids resembled those of the kidneys. Within U-iKOs, the podocytes had typical macronuclei and primary and secondary foot processes. The putative proximal tubule epithelium formed lumen-like structures with dense apical microvilli at the brush border and abundant mitochondria in the cytoplasm. The putative distal tubule epithelium showed sparse and short microvilli and tight junctions (Fig. 6B).

We then implanted the organoids into the CAM and found that they further matured structurally within 7 days (Fig. 6C, Additional file 1: Fig. S5A–C), with glomerular and tubular cells as marked. Finally, we performed confocal microscopy on grafts (Fig. 6D) and showed that glomerular markers PODXL, nephrin, WT1, PAX8, and LAM and tubular markers LTL, CDH1, and PAX8 were present in the developed organoids. We also noticed that PAX8 + parietal epithelial cells were present on the wall of Bowman's capsule, indicating that glomeruli acquired a much more mature architecture in the CAM transplantation. Together, these results suggested that a rather comprehensive recapitulation of kidney function could be established in vitro through our reprogramming approaches.

### Generation of kidney disease models with 3D U-iKOs

**Fig. 7:**
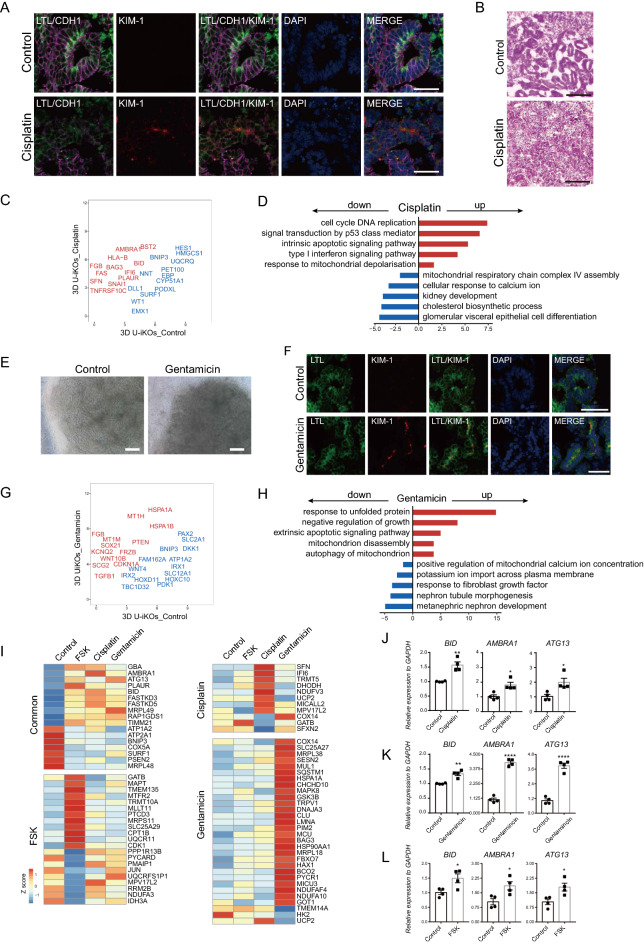
3D U-iKOs disease models. **A** Immunofluorescence analysis of 3D U-iKOs treated with cisplatin (5 μM) for 24 h. Scale bars, 50 μm. **B** H and E staining of U-iKOs treated with cisplatin in A. Scale bars, 100 μm. **C** Scatterplot showing the up-regulated genes (red) and down-regulated genes (blue) between control and cisplatin treatment groups. **D** GO analysis of C. **E** Representative bright-field images of control and gentamicin treatment cells. Scale bars, 250 μm. **F** Immunofluorescence analysis of 3D U-iKOs treated with control and gentamicin (5 mg/mL) for 24 h. **G** Scatterplot showing the up-regulated genes (red) and down-regulated genes (blue) between control and gentamicin treatment groups. **H** GO analysis of G. **I** Heatmap shows common differentially expressed genes of FSK, cisplatin, and gentamicin treatment groups compared to control and their respective differentially expressed genes. **J**–**L** Gene expression analysis for differentially expressed genes after cisplatin, gentamicin, and FSK treatment in I. The relative expression of each transcript to GAPDH expression is presented as the mean ± SEM (n = 4). *P < 0.05 vs. the control group; **P < 0.01 vs. the control group; ****P < 0.0001 vs. the control group

The structure and function of U-iKOs further inspired us to evaluate whether these organoids be used to model kidney diseases. To this end, we treated the U-iKOs with cisplatin for 24 h, which is a nephrotoxic drug used in the chemotherapy of malignant tumors [33]. Interestingly, cisplatin treatment resulted in the up-regulation of kidney injury molecule-1 (KIM-1), a major marker for acute kidney injury (Fig. 7A). Hematoxylin–eosin (HE) staining showed that tubular epithelial structures were nearly destroyed following cisplatin treatment (Fig. 7B). Meanwhile, we collected U-iKOs samples with or without cisplatin treatment for RNA-seq. GO analysis further indicated that cisplatin treatment triggered upregulation of genes that are strongly enriched in cell cycle DNA replication, signal transduction by p53 class mediator, apoptotic signaling pathway, and mitochondrial depolarization while downregulated genes were closely related to glomerular epithelial cells differentiation and mitochondria respiratory chain assembly (Fig. 7C, D). In addition to chemotherapeutic drugs, antibiotics such as gentamicin, are another kind of nephrotoxic drugs [34]. Thus, we treated the U-iKOs with gentamicin for 24 h and observed that U-iKOs displayed similar cellular morphological changes as cisplatin treatment and upregulated KIM-1 expression (Fig. 7E, F). RNA-seq data showed that gentamicin treatment increased the expression of genes involved in unfolded protein, apoptosis, negative regulation of cell growth and mitochondria autophagy, and the downregulated genes related to nephron tubule morphogenesis and ion homeostasis (Fig. 7G, H). On the other hand, forskolin or FSK, a chemical agonist to activate intracellular cAMP levels, is often used for cystogenesis induction in P-iKOs from patients with polycystic kidney disease (PKD) or non-PKD iPSCs [8, 35, 36]. Consistently, we observed that U-iKOs formed enlarged cyst structures after FSK treatment for 4 days, as validated by HE staining and immunostaining with tubule epithelial cell markers (Additional file 1: Fig. S6A–C). Interestingly, we found that FSK treatment enhanced renin, AGT, and PRCP expression which was confirmed in P-iKOs recently [37] (Additional file 1: Fig. S6D, E). Compared with untreated U-iKOs, FSK treatment led to unregulated genes enrichment in the regulation of cell morphogenesis and organelle fission while downregulated genes affected kidney epithelium development and pattern specification process (Additional file 1: Fig. S6F, G). Finally, although all these three models affected mitochondria homeostasis, we assessed and compared the expression of genes involved in mitochondria dynamics. Cisplatin, gentamicin, and FSK treatment all affected mitochondrial function associated genes such as BID, ATG13, BNIP3, AMBRA1, SURF1, and COX5A (Fig. 7I, left), which were previously demonstrated to regulate mitochondria-related kidney pathologies in vivo [3, 38–42]. The expression of BID, AMBRA1, and ATG13 was also validated by qRT-PCR (Fig. 7J–L). On the other hand, mitochondria homeostasis-associated genes were specifically expressed in these three models respectively, suggesting that cisplatin, gentamicin, and FSK treatment resulted in kidney injury in a pathology-specific manner (Fig. 7I, right panel). Then, we also validated differentially altered genes expression in each disease model by qPCR (Additional file 1: Fig. S6H). Taken together, these results demonstrated that U-iKOs can be used as an in vitro model to mimic kidney injury conditions related to mitochondria homeostasis in a personalized manner.

## Discussion

The kidney, one of the highest metabolically active organs in the human body, requires abundant mitochondria to provide sufficient energy to maintain function homeostasis [43]. To the best of our knowledge, this is the first report on the generation of mitochondria-rich kidney organoids via the partial reprogramming of mitochondria-rich human urine cells into the intermediate mesoderm stage. The entire process was induced under the defined medium within 24 days. Furthermore, we developed a defined medium to allow U-iIMPLCs self-renew and possess the potential to generate U-iKOs efficiently in vitro. The U-iKOs could recapitulate kidney structure and function in vitro and mimic mitochondria-related kidney disease conditions through cisplatin, gentamicin, and FSK treatment respectively. Collectively, our study provides a practical platform to study the pathogenesis of kidney diseases, drug screening and regenerative medicine purposes in a personalized manner. Compared to P-iKOs, adult kidney biopsy-derived organoids, and animal models, U-iKOs are appealing for drug toxicity testing and regenerative medicine purposes. The generation of U-iKOs is time-saving and easy to manipulate from both healthy volunteers and patients with CKD of different ages and gender as well as avoids tumorigenicity issues that might occur regarding the application of P-iKOs. In addition, unlike recently reported “tubuloids” which contain only tubular epithelial cells and rely on a conditioned medium, U-iKOs are generated under a defined medium and possess the most typical cell types of kidney lineage, including both epithelial and mesenchymal cells, to form a more complex structure. Thus, U-iKOs resembled an almost complete nephron in vitro and were superior to tubuloids regarding the study of cellular interactions and communications among different kidney lineage cells .

Partial reprogramming of a certain cell type into another bypassing pluripotency is a practical way to induce functional cells in vitro or regenerate and repair tissues in vivo [44–46]. Recently, Chen et al. reported that in situ partial reprogramming of cardiomyocytes into a fetal stage drove heart regeneration in mice [46]. In this report, we selected PAX8 + tubule epithelial cells in hUCs as the starting cells for reprogramming into intermediate mesoderm and kidney lineage cells, which provides a strategy for further optimization of kidney regeneration in situ through partial reprogramming. In addition, partial reprogramming allows initial cells to retain their genetic and epigenetic memory and regain their native phenotypes. Therefore, it is easy for us to generate mitochondria-rich kidney organoids from PAX8 + tubule epithelial cells that contain abundant mitochondria. Previously, we used the same cells to generate iPSCs [13], neural progenitor cells [30], and cardiomyocytes [31], suggesting that hUCs possess high plasticity and are ideal candidates for cell fate transition. We propose a more robust platform to leverage the relative ease of isolating these cells and generate other functional cell types from them for both disease modeling as well as therapy in the future.

Finally, since renal toxicity is a major concern during drug development, a panel of organoids from healthy and renal-compromised individuals may serve as an effective testing platform to evaluate drug safety in the pharmaceutical industry.

## Supplementary Information

**Additional file 1: Fig. S1.** Generation of 2D kidney organoids from hUCs of different donors. (A) Western blot for marker gene of the primitive streak, intermediate mesoderm, nephron progenitor, and segments of the nephron. PAX8 for tubule epithelial and nephron progenitor, CALBINDIN for distal tubule. (B) Gene expression analysis for markers of pluripotency (OCT4 and NANOG), endoderm (SOX17 and FOXA2), and ectoderm (SOX1 and FOX6) during differentiation is presented. Embryonic stem cells (ESCs), defined as endoderm (DE), and neural progenitor cells (NPCs) are shown as the positive control. The relative expression of each transcript to GAPDH expression is presented as the mean ± SEM (n = 6). (C) Volunteer information and 2D U-iKOs induction from urine cells of healthy volunteers and CKD patients. (D) Representative bright-field images for one healthy volunteer (UC-001) and one patient (CKD, PUC-011). Scale bars, 250 μm. (E) Heatmap of markers and signature genes of hUCs, U-iIMPLCs, U-iNPCs, and U-iKOs for the volunteers in B. (F) Immunostaining analysis of primitive streaks marker (T and TBX6) for U-iIMPLCs and nephron progenitor cells maker (PAX2 and SALL1) for U-iNPCs. Scale bars, 50 μm. (G) Comparison of culture conditions of kidney organoid systems in personalized ways. M-iKOs, iPSC-induced organoids with protocol by Morizane; T-iKOs, iPSC-induced organoids with protocol by Takasato. **Fig. S2.** Cell fate changes during U-iKOs differentiation by RNA-seq. (A) Scatterplot showing the differentially expressed genes between hUCs (blue) and U-iIMPLCs (red) in the left panel, U-iIMPLCs (blue) and U-iNPCs (red) in the middle panel, U-iIMPLCs (blue) and U-iKOs (red) in the right panel during differentiation. (B) Gene ontology (GO) analysis for upregulated expressed genes (blue) and downregulated expressed genes of U-iIMPLCs compared to hUCs with p value at the bottom. (C) GO analysis for upregulated expressed genes (blue) and downregulated expressed genes of U-iNPCs compared to U-iIMPLCs with p value at the bottom. (D) GO analysis for upregulated expressed genes (blue) and downregulated expressed genes of U-iKOs compared to U-iIMPLCs with p value at the bottom. **Fig. S3.** ScRNA-seq profiling subpopulations in 3D U-iKOs. (A) Violin plot shows the detected genes, UMI count, and percentage of mitochondrial genes between 3D and 2D U-iKOs. (B) Heatmap indicating Pearson’s correlations on the averaged profiles among cell types between 3D and 2D U-iKOs. (C) UMAP plot of podocyte subcluster in U-iKOs, each dot represents one cell, with color code for each cluster. (D) Heatmap of differentially expressed genes between clusters in C selected signature genes and GO term in the right. (E) UMAP plot of proximal tubule subcluster in U-iKOs, each dot represents one cell, with color code for each cluster. (F) Heatmap of differentially expressed genes between clusters in E selected signature genes and GO term in the right. (G) UMAP plot of the loop of Henle and distal tubule subcluster in U-iKOs, each dot represents one cell, with color code for each cluster. (H) Heatmap of differentially expressed genes between clusters in E selected signature genes and GO term in the right. **Fig. S4.** Characteristics of M-iKOs, T-iKOs, C-iKOs and fetal kidney. (A) Left: GO term of differentially expressed genes in M-iKOs with p value at the bottom. Right: Dotplot shows differentially expressed genes in M-iKOs. (B) Left: GO term of differentially expressed genes in T-iKOs with p value at the bottom. Right: Dotplot shows differentially expressed genes in T-iKOs. (C) Left: GO term of differentially expressed genes in C-iKOs with p value at the bottom. Right: Dotplot shows differentially expressed genes in C-iKOs. (D) Left: GO term of differentially expressed genes in fetal kidney with p value at the bottom. Right: Dotplot shows differentially expressed genes in fetal kidney. (E) Immunostaining analysis of U-iKOs markers for kidney function. LRP2 (megalin), a multi-ligand endocytic receptor; SLC12A1 (NKCC2), a kidney-specific sodium-potassium-chloride cotransporter. Scale bars, 50 μm. **Fig. S5.** Maturing organoids after CAM transplantation. (A) Methodology for the implantation of 3D U-iKOs into CAM. Embryonic day, ED. (B) Macroscopic views of implanted U-iKOs were maintained in CAM for 6 days. (C) H&E staining of implanted U-iKOs implanted in the CAM for 6 days. CAM blood vessels were indicated with black asterisks. Scale bars, 100 μm. (n = 5).** Fig. S6.** 3D U-iKOs after FSK treatment. (A) Representative bright-field images of 3D U-iKOs treated with forskolin (FSK) for 4 days (day 24–28). Scale bars, 250 μm. (B) H&E staining of U-iKOs in the absence (control) or presence of FSK. Scale bars, 100 μm. (C) Immunofluorescence analysis of U-iKOs treated with FSK for 4 days. Scale bars, 50 μm. (D) Gene expression analysis for REN and AGT by RT-qPCR. The relative expression of each transcript to GAPDH expression is presented as the mean ± SEM (n = 4). (E) Gene expression analysis for REN, AGT, and PRCP by RNA-seq. (F) Scatterplot showing the up-regulated genes (red) and down-regulated genes (blue) between control and FSK treatment groups. (G) GO term of the up-regulated genes (red) and down-regulated genes (blue) in the FSK treatment group compared with the control group with p value at the bottom. (H) Gene expression analysis for specifical genes after cisplatin, gentamicin, and FSK treatment in Fig.7I. The relative expression of each transcript to GAPDH expression is presented as the mean ± SEM (n = 4).

**Additional file 2: Table S1.** Antibodies for immunostaining and western blot. **Table S2.** Primer list for RT-qPCR.

## Data Availability

The accession number for the RNA-seq, ATAC-seq, and scRNA-seq data reported in this study is NCBI GEO: GSE198844.

## Abbreviations

hUCs: Human urine cells
U-iIMPLCs: HUCs induced intermediate mesoderm progenitor like cells
U-iKOs: HUCs induced kidney organoids
CKD: Chronic kidney disease
PSCs: Pluripotent stem cells
ESCs: Embryonic stem cells
iPSCs: Induced PSCs
P-iKOs: PSCs-induced kidney organoids
REGM: Renal epithelial growth medium
PODXL: Podocalyxin
ALI: Air–liquid interface
FSK: Forskolin
CAM: Chick chorioallantoic membrane
ED: Embryonic day
RT: Room temperature
TEM: Transmission electron microscopy
qRT-PCR: Quantitative RT-PCR
scRNA-seq: Single cell RNA-seq
TPM: Transcripts per kilobase million
PCA: Principal component analysis
GO: Gene ontology
UMIs: Unique molecular identifiers
HVGs: Highly variable genes
CCA: Canonical correlation analysis
NPCs: Neural progenitor cells
CO: Close to open
OC: Open to close
KIM-1: Kidney injury molecule-1
HE: Hematoxylin–eosin
PKD: Polycystic kidney disease
