# Supplementary figures and images for: Generation of mitochondria-rich kidney organoids from expandable intermediate mesoderm progenitors reprogrammed from human urine cells under defined medium
Source: Cell Biosci. 2022 Oct 15;12:174. doi: 10.1186/s13578-022-00909-0 (PMC9569036; doi:10.1186/s13578-022-00909-0)

Figure S1

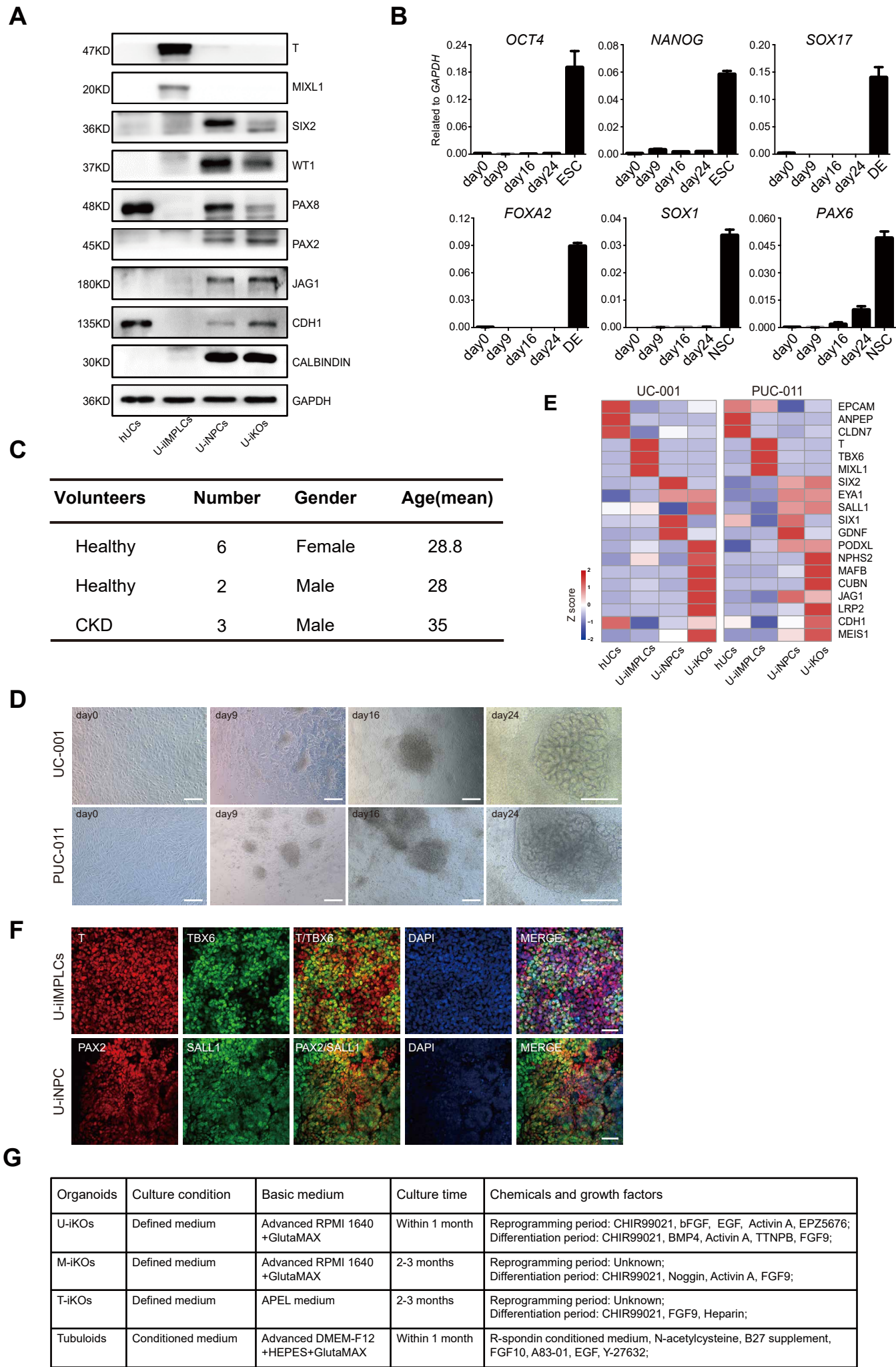

Figure S2

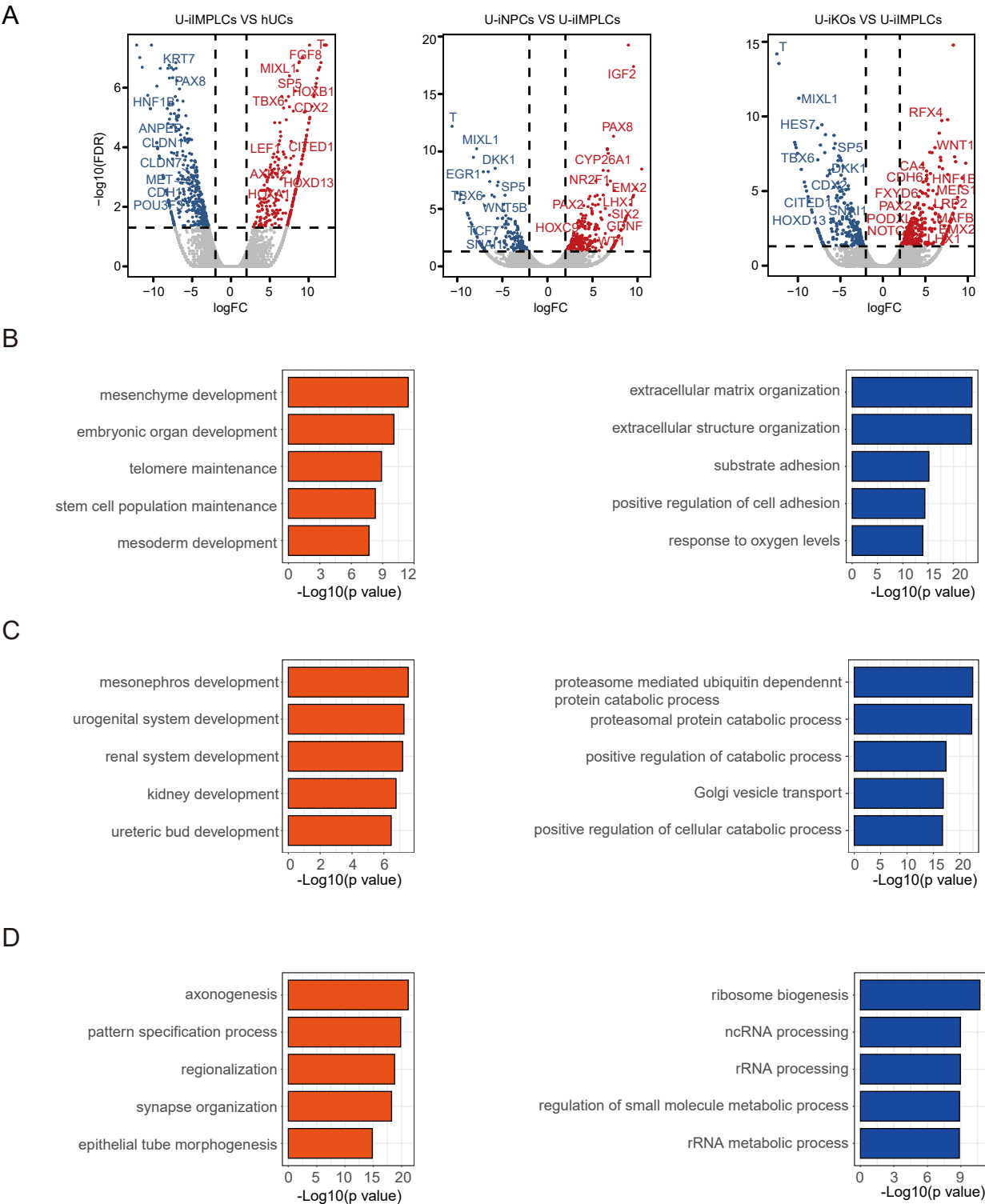

**Figure S3**

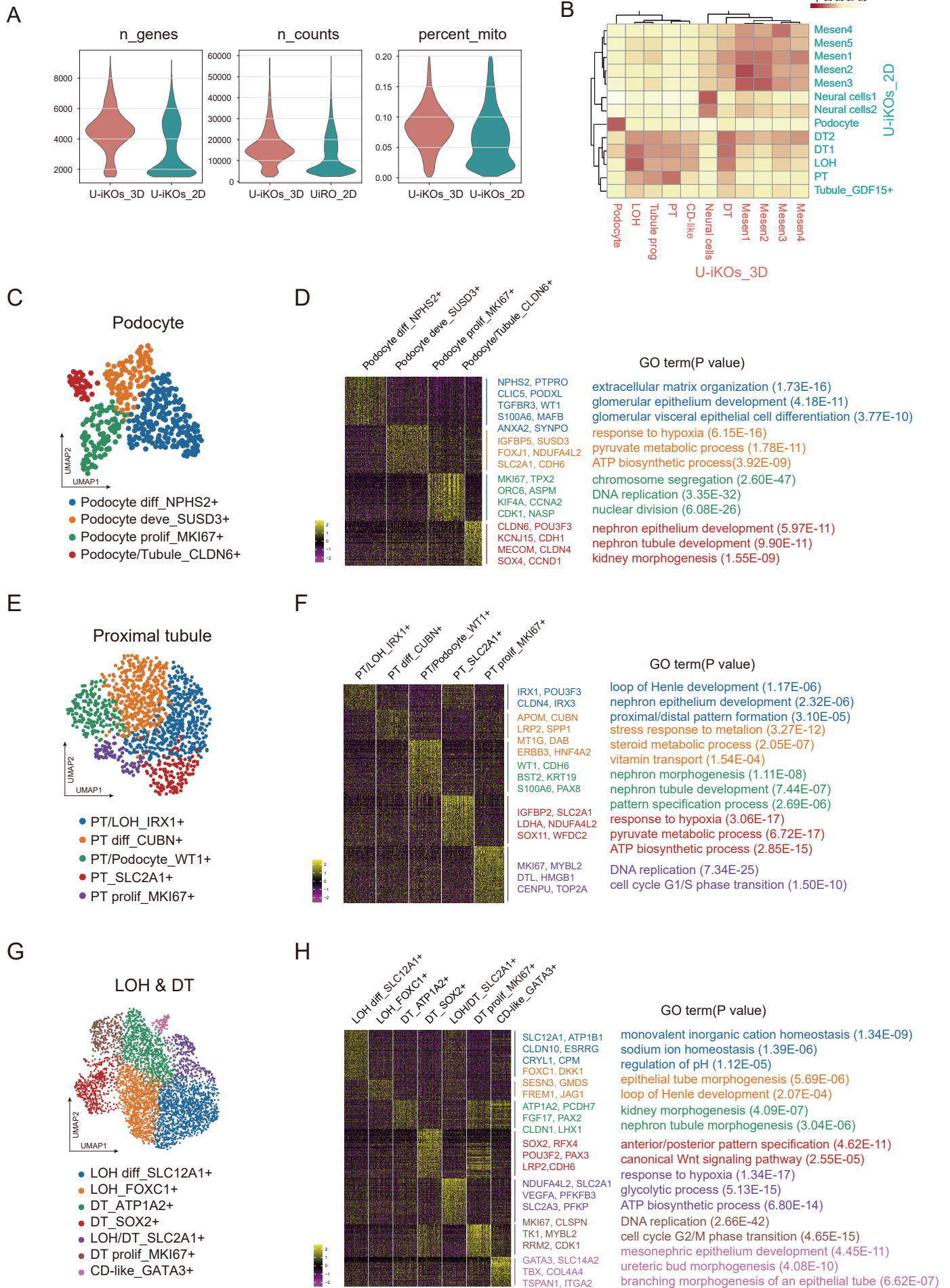

Figure S4

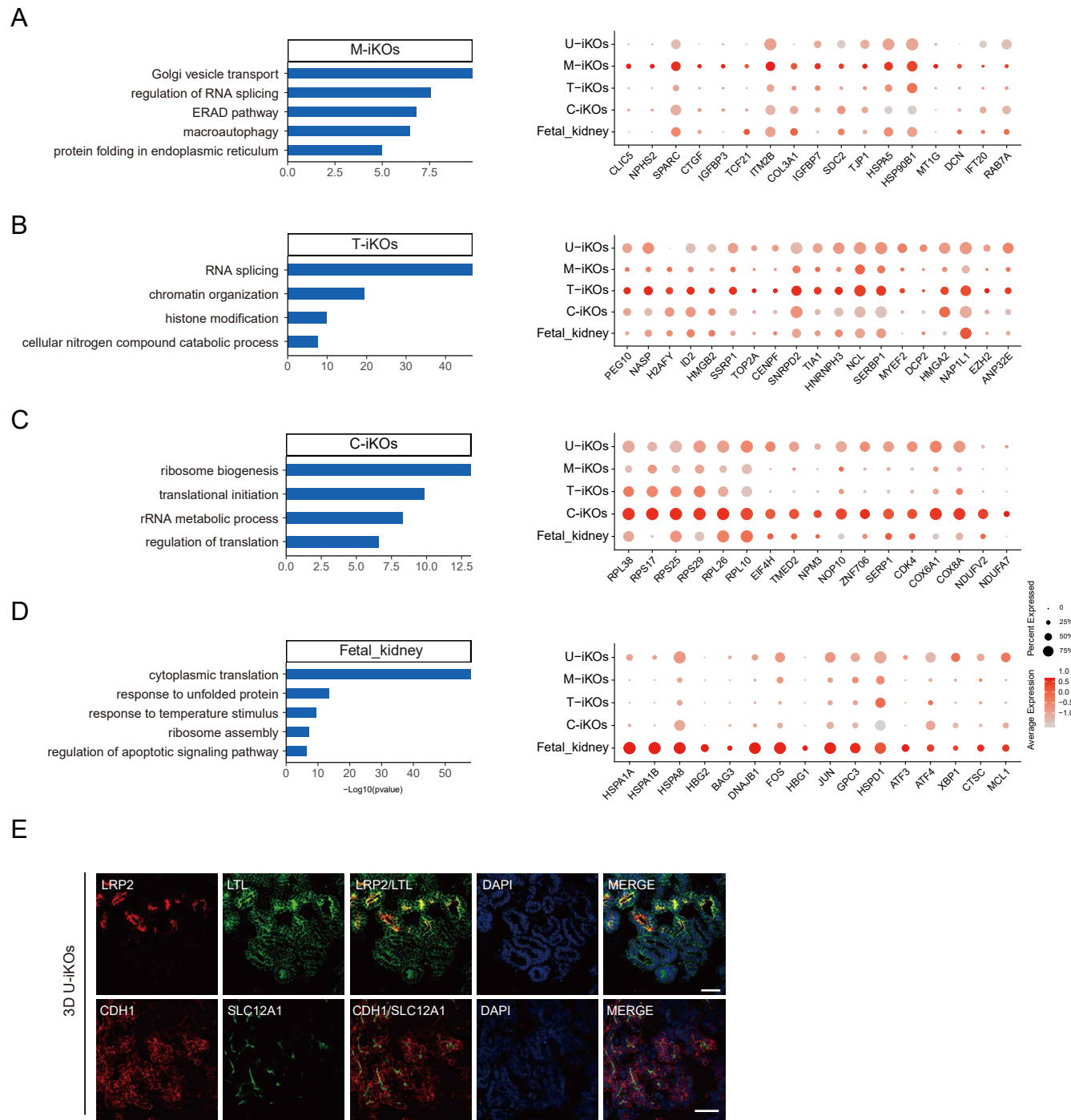

Figure S5

A

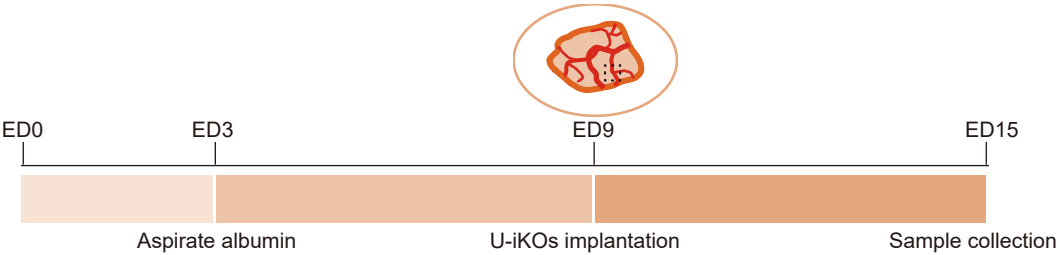

B

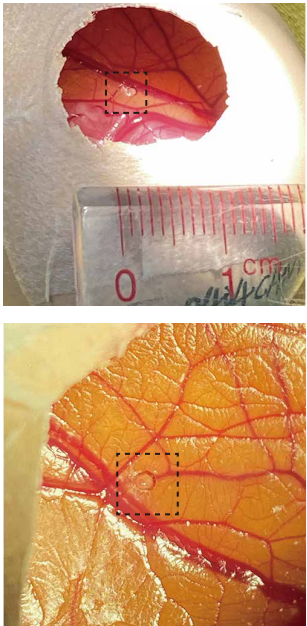

C

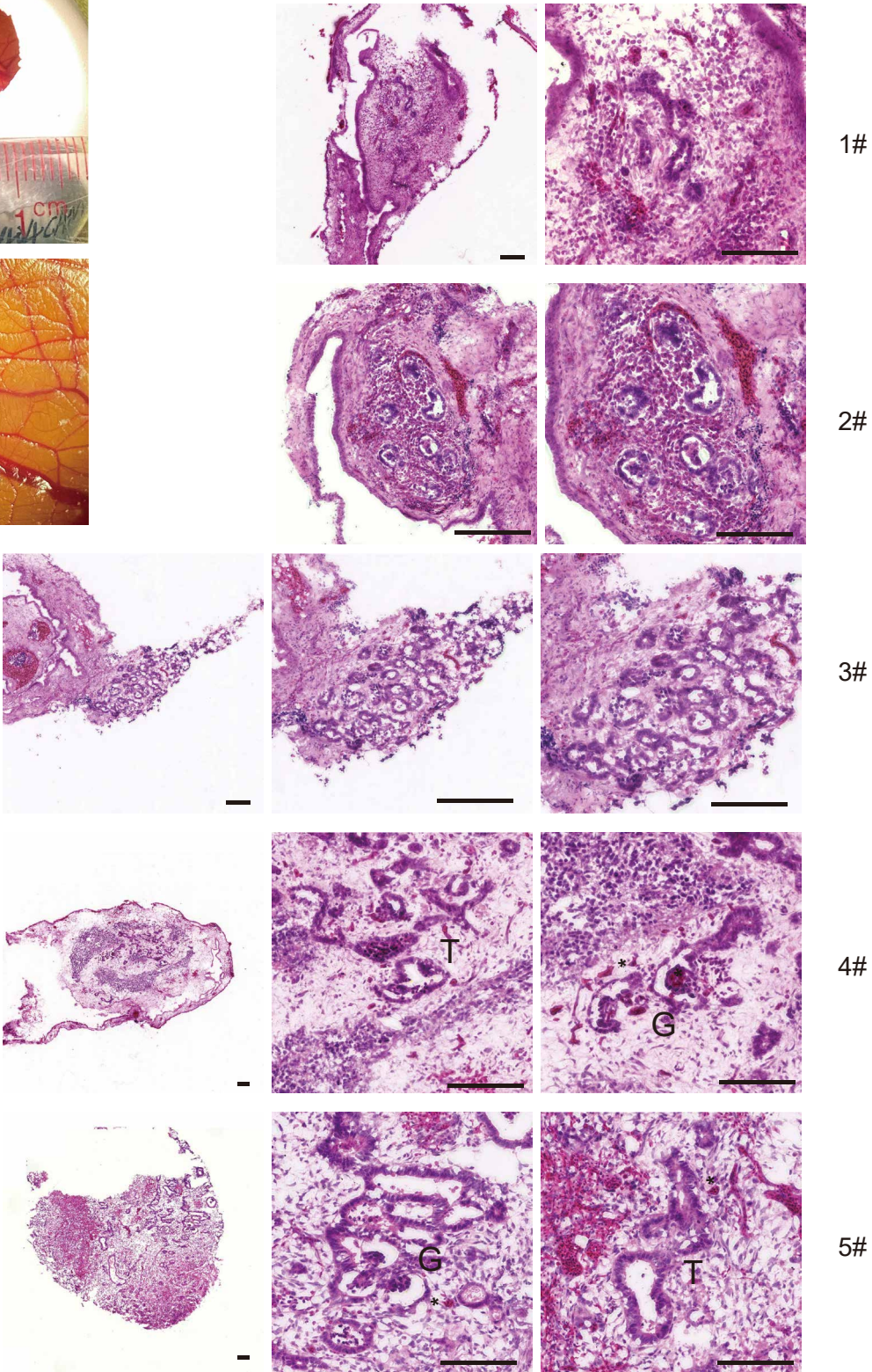

Figure S6

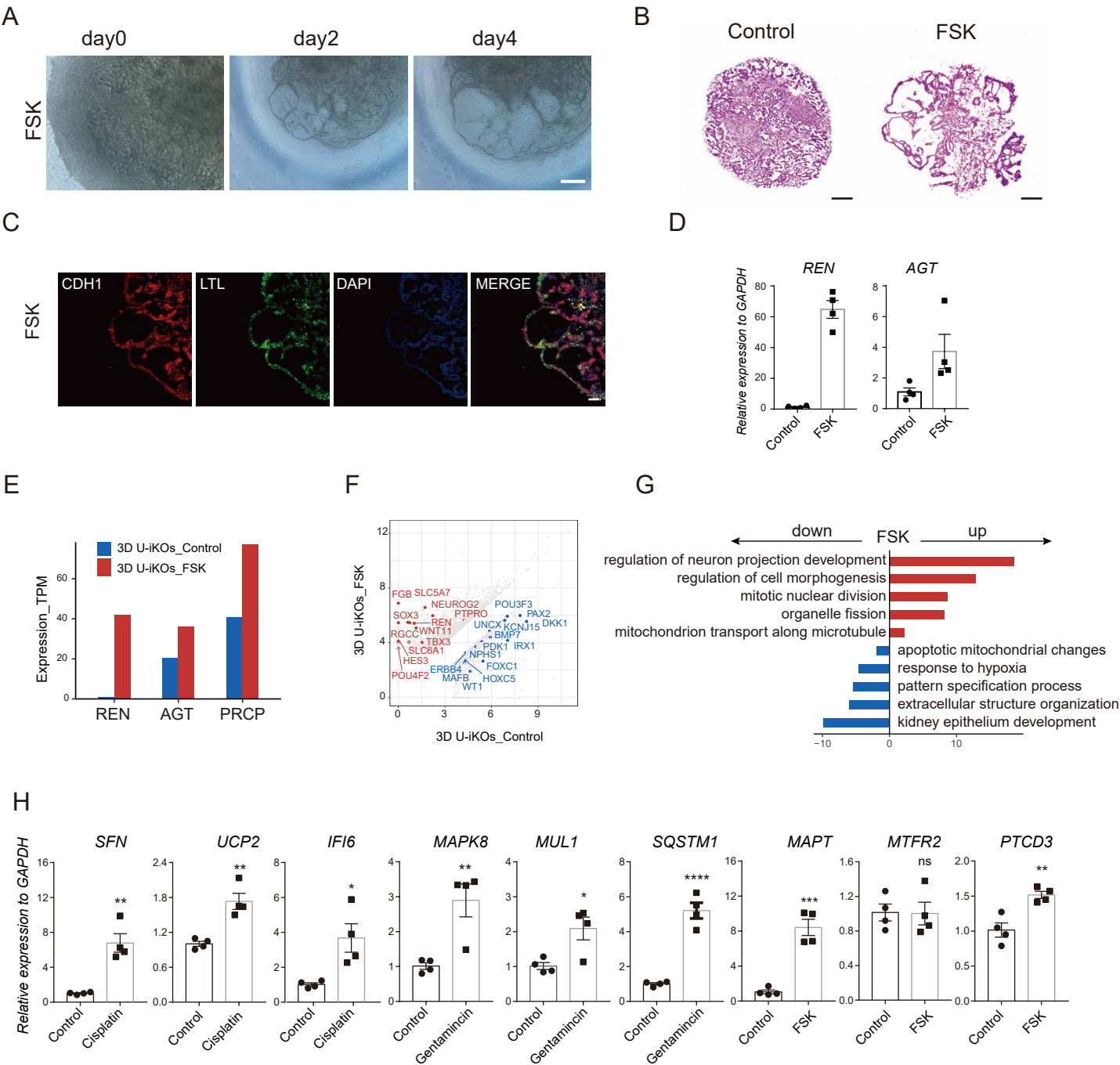

Supplement: Supplementary file 1 — Additional file 1: Fig. S1. Generation of 2D kidney organoids from hUCs of different donors. (A) Western blot for marker gene of the primitive streak, intermediate mesoderm, nephron progenitor, and segments of the nephron. PAX8 for tubule epithelial and nephron progenitor, CALBINDIN for distal tubule. (B) Gene expression analysis for markers of pluripotency (OCT4 and NANOG), endoderm (SOX17 and FOXA2), and ectoderm (SOX1 and FOX6) during differentiation is presented. Embryonic stem cells (ESCs), defined as endoderm (DE), and neural progenitor cells (NPCs) are shown as the positive control. The relative expression of each transcript to GAPDH expression is presented as the mean ± SEM (n = 6). (C) Volunteer information and 2D U-iKOs induction from urine cells of healthy volunteers and CKD patients. (D) Representative bright-field images for one healthy volunteer (UC-001) and one patient (CKD, PUC-011). Scale bars, 250 μm. (E) Heatmap of markers and signature genes of hUCs, U-iIMPLCs, U-iNPCs, and U-iKOs for the volunteers in B. (F) Immunostaining analysis of primitive streaks marker (T and TBX6) for U-iIMPLCs and nephron progenitor cells maker (PAX2 and SALL1) for U-iNPCs. Scale bars, 50 μm. (G) Comparison of culture conditions of kidney organoid systems in personalized ways. M-iKOs, iPSC-induced organoids with protocol by Morizane; T-iKOs, iPSC-induced organoids with protocol by Takasato. Fig. S2. Cell fate changes during U-iKOs differentiation by RNA-seq. (A) Scatterplot showing the differentially expressed genes between hUCs (blue) and U-iIMPLCs (red) in the left panel, U-iIMPLCs (blue) and U-iNPCs (red) in the middle panel, U-iIMPLCs (blue) and U-iKOs (red) in the right panel during differentiation. (B) Gene ontology (GO) analysis for upregulated expressed genes (blue) and downregulated expressed genes of U-iIMPLCs compared to hUCs with p value at the bottom. (C) GO analysis for upregulated expressed genes (blue) and downregulated expressed genes of U-iNPCs compare [file 13578_2022_909_MOESM1_ESM.pdf]
