# Supplementary material for: Generation of mitochondria-rich kidney organoids from expandable intermediate mesoderm progenitors reprogrammed from human urine cells under defined medium
Source: Cell Biosci. 2022 Oct 15;12:174. doi: 10.1186/s13578-022-00909-0 (PMC9569036; doi:10.1186/s13578-022-00909-0)
Supplement: Supplementary file 2 — Additional file 2: Table S1. Antibodies for immunostaining and western blot. Table S2. Primer list for RT-qPCR. [file 13578_2022_909_MOESM2_ESM.docx]

**Supplementary file 2**

**Table S1. Antibodies** **for immunostaining and western blot**

| **Antibodies** | **Source** | **Identifier** |
| --- | --- | --- |
| T | Abcam | Cat. #ab209665 |
| MIXL1 | Proteintech | Cat. #22772-1-AP |
| SALL1 | R&D | Cat. #PP-K9814-00 |
| PAX2 | Biolegend | Cat. #901001 |
| Lotus Tetragonolobus Lectin (LTL), Fluorescein labeled | Vector Laboratories | Cat. #FL-1321-2 |
| CDH1 | Abcam | Cat. #ab11512-100 |
| WT1 | Abcam | Cat. #ab89901 |
| SIX2 | Proteintech | Cat. #11562-1-AP |
| PODXL | R&D | Cat. #AF1658 |
| CALBINDIN | Proteintech | Cat. #14479-1-AP |
| PAX8 | Proteintech | Cat. #10336-1-AP |
| LAM (Laminin) | sigma | Cat. #L9393 |
| JAG1 | R&D | Cat. #AF1277 |
| GAPDH | Proteintech | Cat. #600041-lg |
| Nephrin | R&D | Cat#AF4269 |
| KIM-1 | R&D | Cat. #AF1750 |
| SLC12A1 | Proteintech | Cat. #18970-1-AP |
| PNA | Vector Laboratories | Cat. #FL-1071 |
| LRP2 | Proteintech | Cat. #19700-1-AP |
| HRP-conjugated Goat Anti-Rabbit lgG | Proteintech | Cat. #SA00001-1 |
| HRP-conjugated Goat Anti-Mouse lgG | Proteintech | Cat. #SA00001-2 |
| HRP-conjugated Donkey Anti-Goat lgG | Proteintech | Cat. #SA00001-3 |
| HRP-conjugated Rabbit Anti-Sheep lgG | Proteintech | Cat. #SA00001-16 |
| Donkey anti-Mouse lgG Alexa Fluor 488 | Invitrogen | Cat. #A-21202 |
| Donkey anti-Goat lgG, Alexa Fluor 488 | Invitrogen | Cat. #A-11055 |
| Donkey anti-Rat lgG Alexa Fluor 594 | Invitrogen | Cat. #A-21209 |
| Donkey anti-sheep, Alexa Fluor 594 | Invitrogen | Cat. #A11016 |
| Goat anti-rabbit lgG, Alexa Fluor 568 | Invitrogen | Cat. #A11011 |
| Goat anti-mouse lgG, Alexa Fluor 568 | Invitrogen | Cat. #A11004 |
| Goat anti-rabbit lgG, Alexa Fluor 488 | Invitrogen | Cat. #A11008 |
| Goat anti-mouse lgG, Alexa Fluor 488 | Invitrogen | Cat. #A11001 |
| Donkey anti-Rabbit IgG, Alexa Fluor 594 | Invitrogen | Cat. #A-21207 |
| Chicken anti-Rabbit IgG, Alexa Fluor 647 | Invitrogen | Cat. #A-21443 |
| Donkey anti-Mouse IgG, Alexa Fluor 647 | Invitrogen | Cat. #A-31571 |

**Table S2. Primer list** **for RT-qPCR**

| **Gene** | **Forward primer** | **Reward primer** |
| --- | --- | --- |
| GAPDH | GTGGACCTGACCTGCCGTCT | GGAGGAGTGGGTGTCGCTGT |
| T | TGCTTCCCTGAGACCCAGTT | GATCACTTCTTTCCTTTGCATCAAG |
| MIXL1 | AGGCAGGAGAATCACTTG | GGCTACCACAGAACCATAG |
| TBX6 | GTGACAGCCTACCAGAAC | TCGCTCCCTCTTACAGTT |
| SIX2 | GTCAGCAACTGGTTCAAG | TGGATGATGAGTGGTCTG |
| WT1 | AAGGACTGTGAACGAAGG | TTGTGATGGCGGACTAAT |
| HOXD11 | TGGATGAAAGTGAAGAGGAA | TTCTGTCAGTTGCTTGGT |
| PAX2 | CCCAAAGTGGTGGACAAGAT | GAAAGGCTGCTGAACTTTGG |
| CDH6 | ATGAGAACTTACCGCTACTT | AGAACTGATTCCACATCCA |
| JAG1 | TCTTACTACGGAGCACATT | CGCCTCTGAACTCTTACT |
| PODXL | TCCCAGAATGCAACCCAGAC | GGTGAGTCACTGGATACACCAA |
| OSR1 | TCCTACAACCTACTTATCCAT | CCACACTCTTGACACTTG |
| CUBN | TCATTCTCCTCAACTTCACT | TGCTACCAATCTCAACATAATC |
| LRP2 | CTCACCTCTTGCCTGTAA | ATGCCACTTCGGATAACT |
| SLC12A1 | TTGGCGTGGTTATAGTCA | GGCGTTGTCTTAGTAATGTT |
| AGT | GTTGCTGCTGAGAAGATTG | AGTCACCGAGAAGTTGTC |
| ATP1A2 | GCCTCCTCATCTTCATCTAT | TCAGTAGTATGTCTCCTTCTC |
| MUC1 | TGGAGACACAGTTCAATCA | GGCAATGAGATAGACAATGG |
| MRPL48 | CCTGGACTCAGTGCTTAC | CTTCAGTGTGCTCCTTCA |
| COX7A2L | CAGAAGCACCACCTATCA | TTTCCCAGCATAATCATACAC |
| BID | CTAGAGACATGGAGAAGGAG | GTGCGTAGGTTCTGGTTA |
| AMBRA1 | TCTTCACTGTCCATTCCAA | TCTCTGTCTGTCCTCCAT |
| ATG13 | GTCATTGCTGCTGAAGTC | AACACGAACTGTCTGGAA |
| OCT4 | CCTCACTTCACTGCACTGTA | CAGGTTTTCTTTCCCTAGCT |
| SOX2 | CCCAGCAGACTTCACATGT | CCTCCCATTTCCCTCGTTTT |
| NANOG | AAGGTCCCGGTCAAGAAACAG | CTTCTGCGTCACACCATTGC |
| SOX17 | ACCGCACGGAATTTGAAC | GCAGTAATATACCGCGGAGC |
| FOXA2 | ACAGCAGTCTTCTTCACC | AGCAGGAGTCTACACAGTA |
| SOX1 | TTTCCCCTCGCTTTCTCA | TGCAGGCTGAATTCGGTT |
| PAX6 | TTGCTTGGGAAATCCGAG | TGCCCGTTCAACATCCTT |
| REN | TCCACTATATCAACCTCATCA | TTACACTTCACGACATAATCAA |
| AGT | GTTGCTGCTGAGAAGATTG | AGTCACCGAGAAGTTGTC |
| IFI6 | TCCTCATCCTCCTCACTAT | TGTCCAGTATTGAGCAGAA |
| SFN | TGTCCAGTATTGAGCAGAA | CAGGTAGCGGTAGTAGTC |
| UCP2 | TGTGGTAAAGGTCCGATT | TGGTCTTGTAGGCATTGA |
| MAPK8 | TTATGGACTTGGAGGAGAG | ACAGACGACGATGATGAT |
| MUL1 | CGTGCCTTATGCTGTTATAG | GTGTTGGTCCTCTGATGA |
| SQSTM1 | GGAGCAGATGAGGAAGAT | TGGAGTTCACCTGTAGAC |
| MAPT | TCCACTGAGAACCTGAAG | CCTAATGAGCCACACTTG |
| PTCD3 | TTGATTGAAGCAACAGTA | GGCGAATAATATGGTGAT |
| MTFR2 | ACTCTGTAGACTCTGATAA | ATAACTGGCTTCTTCATC |
